# Prognostic Nutritional Index and Cancer Prognostic Outcomes: An Umbrella Review of Systematic Reviews and Meta-analyses of Observational Studies

**DOI:** 10.1016/j.advnut.2026.100641

**Published:** 2026-04-17

**Authors:** Zhi-Tong Li, Ming-Li Sun, Fang-Hua Liu, Yu Li, Rui-Han Bao, Xiao-Feng Jiang, Jun Liu, Zhao Lu, Ying Yang, Yong Yu, Na Zhao, Qian Chen, Qian Fang, Zan-Fei Feng, Xian-Xian Jia, Dong-Run Li, Meng Luan, Jing Ma, Ying Wang, Yi-Lin Xu, Dan Yang, Miao Yang, Rui Yang, Xue-Li Bai, Jing Yang, Tie-Gang Li, Ting-Ting Gong, Qi-Jun Wu

**Affiliations:** 1 Department of Cardiology, Shengjing Hospital of China Medical University, Shenyang, Liaoning, China; 2 Department of Obstetrics and Gynecology, Shengjing Hospital of China Medical University, Shenyang, Liaoning, China; 3 College of Clinical Medicine for Obstetrics & Gynecology and Pediatrics, Fujian Medical University, Fuzhou, China; 4 Department of Epidemiology, School of Public Health, China Medical University, Shenyang, Liaoning, China; 5 Key Laboratory of Environmental Stress and Chronic Disease Control & Prevention, Ministry of Education, China Medical University, Shenyang, Liaoning, China; 6 Department of Obstetrics and Gynecology, The Fourth Affiliated Hospital of China Medical University, Shenyang, Liaoning, China; 7 Department of Cardiology, The Fourth Affiliated Hospital of China Medical University, Shenyang, Liaoning, China; 8 Department of Radiology, Shengjing Hospital of China Medical University, Shenyang, Liaoning, China; 9 Department of Hematology, Shengjing Hospital of China Medical University, Shenyang, Liaoning, China; 10 Department of Ophthalmology, Shengjing Hospital of China Medical University, Shenyang, Liaoning, China; 11 Department of Laboratory Medicine, The Fourth Affiliated Hospital of China Medical University, Shenyang, China; 12 Department of Pediatrics, Shengjing Hospital of China Medical University, Shenyang, Liaoning, China; 13 Department of Endocrinology, The Fourth Affiliated Hospital of China Medical University, Shenyang, Liaoning, China; 14 Department of Emergency Medicine, Shengjing Hospital of China Medical University, Shenyang, Liaoning, China; 15 Department of Obstetrics and Gynecology, The Affiliated Reproductive Hospital of China Medical University, Shenyang, Liaoning, China

**Keywords:** cancer, observational study, prognostic nutritional index, prognostic outcomes, umbrella review

## Abstract

Prognostic nutritional index (PNI) has been widely investigated as a predictor of outcomes in patients with cancer; however, a comprehensive synthesis evaluating the breadth, methodological quality, and certainty of this evidence is lacking. We aimed to systematically review and critically appraise existing meta-analyses on the association between PNI and cancer prognostic outcomes. We conducted a comprehensive search of PubMed, Embase, Web of Science, and the Cochrane Database of Systematic Reviews from database inception through 28 February, 2025. Methodological quality of included systematic reviews and meta-analyses was assessed using A Measurement Tool to Assess Systematic Reviews (AMSTAR), and the certainty of evidence was graded according to the Grading of Recommendations, Assessment, Development and Evaluation (GRADE) framework. The protocol was registered prospectively on the International Platform of Registered Systematic Review and Meta-analysis Protocols platform (INPLASY2025110096). A total of 64 systematic reviews and meta-analyses, comprising 265 quantitative syntheses, were included. Of these, 224 (84.5%) reported statistically significant quantitative syntheses (*P* < 0.05) under random-effects models. AMSTAR assessment classified 44 (68.8%) systematic reviews and meta-analyses as high quality. GRADE evaluation indicated that 121 (45.7%) quantitative syntheses were supported by high-certainty evidence. Elevated PNI was consistently associated with improved overall survival (OS) across both pan-cancer and cancer-specific analyses. In pan-cancer populations, higher PNI was significantly associated with longer OS, prolonged progression-free survival, and higher objective response rates. Significant prognostic benefits of elevated PNI for OS were also observed in specific malignancies, including pancreatic cancer, gastric cancer, advanced-stage lung cancer, and glioma. Although a higher PNI is robustly associated with favorable prognostic outcomes in diverse cancers, marked heterogeneity in PNI cutoff values across studies underscores the urgent need for standardized, population-specific thresholds validated through prospective diagnostic accuracy studies.

**Statement of significance**

This UR offers a comprehensive and up-to-date synthesis of the evidence linking the PNI with cancer outcomes across diverse malignancies. It critically evaluates the strength, consistency, and certainty of quantitative syntheses between PNI and key prognostic endpoints—including overall survival, progression-free survival, and treatment response—and highlights the potential of PNI as a readily available, cost-effective biomarker for risk stratification in oncology practice.

## Introduction

The global burden of cancer is escalating due to an aging and growing population, coupled with a rising demand for straightforward and efficient predictive indicators [1]. Currently, pathological classification, staging, molecular markers, and patients’ physical conditions are commonly employed to evaluate cancer prognostic outcomes. Nevertheless, people with cancer sharing the same tumor–node–metastasis (TNM) stage and histological subtype frequently exhibit divergent prognoses [2]. Moreover, the detection of genetic biomarkers poses significant financial and logistical challenges, particularly for people in developing countries. This underscores the urgent need for simple, affordable, and robust indicators to predict prognostic outcomes of people with cancer, thereby facilitating the enhancement of personalized cancer treatment strategies.

Accumulating evidence indicates that cancer prognosis is shaped not only by tumor-related factors but also by the host’s nutritional and immune status [[3], [4], [5], [6]]. The prognostic nutritional index (PNI), originally proposed by Buzby et al. [7], has emerged as a promising composite marker. It is computed from serum albumin concentration and total lymphocyte count, thus integrating both nutritional and immunological aspects. It integrates serum albumin concentrations and peripheral blood lymphocyte counts into a single score using the formula: 10 × albumin concentration (grams per deciliter) + 0.005 × total lymphocyte count. Both parameters are readily available in routine laboratory tests, making PNI practical for widespread use. Although guidelines do not explicitly recommend PNI, PNI has been widely used in clinical practice for prognosis assessment of people with cancer. Recent meta-analyses have highlighted the potential of PNI as a prognostic indicator across diverse cancer types [8,9], such as gastric carcinoma [10], lung carcinoma [11], and glioma [12], but the results have not been consistent. For instance, one meta-analysis reported a significant association between higher PNI and prolonged disease-free survival (DFS) in people with cervical cancer [13], whereas another found no statistically significant association between PNI strata and DFS in the same population [14].

The umbrella review (UR) methodology serves as a standardized tool to offer a relatively comprehensive overview of recently published systematic reviews and meta-analyses on a specific topic [[15], [16], [17]]. This methodology offers a standardized approach to synthesizing existing evidence, enabling the assessment of quality, comparison of findings, and evaluation of the robustness of conclusions across multiple reviews [17,18]. In this study, we conducted an UR of systematic reviews and meta-analyses of observational studies to evaluate the association between the PNI and cancer-related prognostic outcomes among individuals diagnosed with malignant tumors.

## Methods

The UR was formulated in strict adherence to the PRISMA guidelines [19]. The protocol for this UR has been prospectively registered in the International Platform of Registered Systematic Review and Meta-analysis Protocols (registration number: 2025110096).

### Search strategy

A systematic search was conducted across PubMed, Embase, Web of Science, and the Cochrane Database of Systematic Reviews to identify systematic reviews and meta-analyses investigating the association between the PNI and cancer prognostic outcomes. The search covered the databases from inception until 28 February, 2025. The detailed search strategy, including keywords and Boolean operators, is presented in Supplementary Table 1. To identify additional relevant systematic reviews and meta-analyses, we manually reviewed the reference lists of included reviews, as well as the bibliographies of key systematic reviews and meta-analyses.

### Inclusion criteria and exclusion criteria

Two experienced reviewers (Z-TL and M-LS) independently screened the titles and abstracts of retrieved systematic reviews and meta-analyses without language restrictions. Full-text assessment was then performed to assess study eligibility according to prespecified inclusion criteria. Disagreements arising during the screening process were resolved through consensus or consultation with a third reviewer (Q-JW). Studies were included in accordance with the PECOS (Participants, Exposure, Comparison, Outcome, and Study design) framework:

1) Participants: individuals diagnosed with malignant tumors.
2) Exposure and comparison: different groups of the PNI.
3) Outcomes: any cancer prognostic outcomes, including overall survival (OS), DFS, recurrence-free survival (RFS), and progression-free survival (PFS).
4) Study design: systematic reviews and meta-analyses of observational studies, encompassing cohort, case-control, and cross-sectional designs.

We excluded narrative reviews, systematic reviews without quantitative synthesis, studies lacking sufficient data or relevant outcomes, and a small number of studies with same exposure and same outcome.

Whenever >1 meta-analysis addressed the same scientific question, the one with the largest number of included primary studies was retained for the main analysis [20]. Effect estimates from systematic reviews and meta-analyses based on <3 primary studies were excluded [21]. If a given outcome was analyzed using >1 comparative approach (e.g., lower compared with higher, higher compared with lower, etc.), all comparative forms were retained in our UR [22].

### Data extraction

Two experienced reviewers (Z-TL and M-LS) independently extracted relevant data from each included systematic reviews and meta-analyses. Any discrepancies were resolved through discussion with a senior reviewer (Q-JW). The following information was extracted from each eligible systematic review and meta-analysis: first author, publication year, database, retrieval date, study design (e.g., prospective cohort, retrospective cohort, case-control), number of included primary studies, participant number, comparison groups, cancer prognostic outcome type, cancer site, and meta-analysis metrics [e.g., hazard ratio (HR), relative risk] with their corresponding 95% confidence interval (CI). For primary studies included in the systematic reviews and meta-analyses, we also extracted the first author, publication year, participant number, statistical metrics, and 95% CI.

### Assessment of methodological quality

The methodological quality of the included systematic reviews and meta-analyses was evaluated using A Measurement Tool to Assess Systematic Reviews (AMSTAR) [23], which evaluates 11 domains: literature search strategy, study selection process, data extraction methods, statistical analysis approach, and bias assessment [24]. The AMSTAR score was classified as high (8–11), moderate (4–7), and low (0–3). Two trained reviewers (Z-TL and M-LS) independently conducted the quality assessment of methodologies. Discrepancies were resolved through discussion with a senior reviewer (Q-JW).

### Data analysis

For each identified meta-analysis, we retrieved data from its constituent primary studies and recalculated summary effect sizes using random-effects models, along with their 95% CI [17,25]. Heterogeneity was assessed in each meta-analysis using the *I*^*2*^ statistic, where an *I*^*2*^ value >50% or >75% indicated moderate or high heterogeneity, respectively [26].

### Sensitivity analyses

Sensitivity analysis was performed to verify the robustness of our findings by excluding primary studies with small samples (studies with sample sizes falling below the 25th percentile of the distribution within that specific meta-analysis) or high risk of bias [27]. We further list systematic reviews lacking quantitative synthesis to ensure that any unique outcome was considered. All statistical analyses were performed using STATA version 16.0 (StataCorp LLC, College Station, Texas, USA).

### Assessment of evidence certainty

In accordance with Grading of Recommendations, Assessment, Development and Evaluation (GRADE) framework, the certainty of evidence was categorized as high, moderate, low, or very low [28]. In prognostic systematic reviews, the best available evidence commonly derives from cohort studies, because randomized controlled trials are rarely applicable for assessing prognostic factors. Following current GRADE guidance for prognostic evidence, the initial certainty of findings is considered high, even when based on observational studies, provided the studies are methodologically robust and directly address the prognostic association [29]. The evidence certainty may be downgraded due to factors such as risk of bias, inconsistency among results, indirectness, imprecision, and publication bias. Risk of bias was assigned when primary studies with high risk of bias constituted >50% of the total weight. The Newcastle–Ottawa Scale tool was the primary tool for assessing the risk of bias in the included primary studies. Imprecision was identified when the sample size was inadequate (<400). Indirectness was evaluated based on discrepancies in study populations, exposure, comparison, or the measurement of outcome. Inconsistency was assigned when heterogeneity, as measured by the *I*^*2*^ statistic, exceeded 50% indicating moderate heterogeneity. Publication bias was deemed present if the funnel plot exhibited asymmetry or if the *P* value for Egger’s test or Begg’s test was < 0.05 [30]. Conversely, evidence certainty could be upgraded for reasons such as a substantial effect size, the presence of a dose–response relationship, or the existence of plausible residual confounding that might underestimate the true effect [30,31].

## Results

### Literature review

**FIGURE 1:**
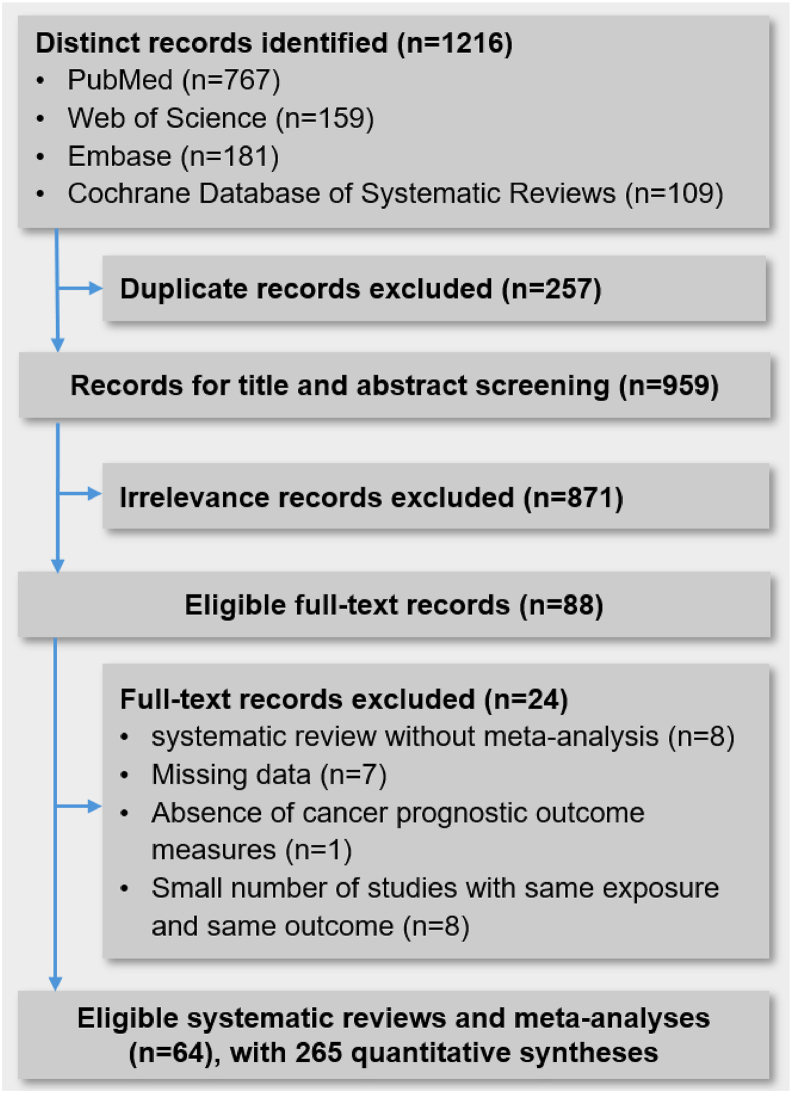
Flow diagram of the study selection process.

The initial database search yielded 1216 records across PubMed, Embase, Web of Science, and the Cochrane Database of Systematic Reviews. After deduplication, 959 unique records remained. Following title and abstract screening, 871 records were excluded. Full-text evaluation led to the exclusion of an additional 24 records due to predefined criteria (Supplementary Table 2). Ultimately, 64 systematic reviews and meta-analyses comprising 265 quantitative syntheses [8], [9], [10], [11], [12], [13], [14],[32], [33], [34], [35], [36], [37], [38], [39], [40], [41], [42], [43], [44], [45], [46], [47], [48], [49], [50], [51], [52], [53], [54], [55], [56], [57], [58], [59], [60], [61], [62], [63], [64], [65], [66], [67], [68], [69], [70], [71], [72], [73], [74], [75], [76], [77], [78], [79], [80], [81], [82], [83], [84], [85], [86], [87], [88] were eligible to be included in the present UR (Figure 1).

### Study characteristics

The eligible 64 systematic reviews and meta-analyses published from 2014 to 2025 (Table 1), which estimated PNI with kinds of cancers, including digestive system cancers (*n =* 80, 30.1%), respiratory system cancers (*n =* 21, 7.9%), urinary system cancers (*n =* 39, 14.7%), reproductive system cancers (*n =* 65, 24.5%), nervous system cancers (*n =* 8, 3.0%), head and neck cancers (*n =* 22, 8.3%), hematologic system cancers (*n =* 2, 0.8%), breast cancers (*n =* 2, 0.8%), and other cancers (*n =* 26, 9.9%). Eleven types of cancer prognostic outcomes, encompassing OS, DFS, RFS, PFS, distant metastasis-free survival, cancer-specific survival (CSS), objective response rate, disease control rate, mortality, rate of adverse events, and postoperative complications. OS was the most frequently assessed endpoint, constituting the primary outcome in many meta-analyses (*n =* 156, 60.9%). We observed that the median number of primary studies was 12 (range: 6–72), and the median number of participants was 3147 (range: 187–23,970). There were 12 (18.8%) systematic reviews and meta-analyses that included the prospective cohort studies. Besides, the cutoff values used to define high and low PNI were varied across the included reviews, ranging from 31.1 to 57.0 (Supplementary Table 3). Among the 265 quantitative syntheses, the magnitude of the observed summary random-effects estimates ranged from 0.31 to 4.05. A total of 224 meta-analyses (84.5%) showed statistical significance at the *P* < 0.05 level based on random-effects model. Out of the 265 quantitative syntheses, 134 (50.6%) displayed low heterogeneity, 67 (25.3%) exhibited high heterogeneity, and 64 (24.1%) presented very high heterogeneity (Supplementary Table 4).

**TABLE 1:** Characteristics of meta-analyses studying prognostic nutritional index with cancer prognostic outcomes.

Author, year, ref	Cancer site	Outcomes	Searched database	Retrieval deadline	Study design	No. of studies	Total participants	Level of comparison
Digestive system cancers								
Deng et al., 2024 [88]	Gastric cancer	OS/CSS/RFS/postoperative complications	Cochrane Library, PubMed, Embase, Google Scholar, Baidu Scholar	To 20 February, 2022	Retrospective or prospective	38	23,756	Low vs. high
Fan et al., 2019 [55]	Hepatocellular carcinoma	OS/RFS	MEDLINE, Embase, Ovid, Google Scholar, and Cochrane databases	To February 2019	Retrospective cohort	7	187	Low vs. high
Hou et al., 2024 [35]	Gastric or gastro-esophageal junction cancer	objective remission rate, disease control rate/OS/PFS	PubMed, Embase, WOS, SpringerLink, and the Cochrane Library	To 5 December, 2023	Prospective	8	813	High vs. low
Jiang et al., 2021 [67]	Esophageal cancer	OS/DFS/CSS	PubMed, WOS, and Cochrane Library Databases	To March 2020	—	72	22,260	Low vs. high
Kang et al., 2022 [77]	Gastrointestinal stromal tumors	OS/RFS	PubMed, WOS, Embase, and Cochrane Library	To December 2021	Retrospective cohort	8	2307	Low vs. high
Li et al., 2018 (a) [10]	Gastric cancer	OS/CSS/RFS/postoperative complications/mortality	Scopus and PubMed	To 1 June, 2018	Prospective and retrospective cohort	25	14,403	Low vs. high
Li et al., 2019 (a) [49]	Pancreatic cancer	OS	PubMed, Embase, WOS, and Cochrane Library	To 31 December, 2017	Retrospective	10	2064	Low vs. high
Li et al., 2019 (b) [51]	Esophageal squamous cell carcinoma	OS/RFS/CSS	PubMed, Embase, WOS	To 30 April, 2018	Retrospective	9	2276	Low vs. high
Li et al., 2022 (a) [76]	Gastrointestinal stromal tumors	RFS	PubMed, Embase, and the Cochrane Library	To June 2022	Retrospective	8	2627	High vs. low
Liao et al., 2019 [53]	Esophageal cancer	OS/CSS/RFS	PubMed and Embase	To 26 March, 2019	Retrospective	12	3118	Low vs. high
Liu and Li, 2025 [34]	Pancreatic head adenocarcinoma, pancreatic carcinoma, pancreatoduodenectomy, pancreatic head carcinoma, periampullary carcinoma, ampullary carcinoma, ampulla of Vater carcinoma, pancreatic adenocarcinoma, pancreatic ductal adenocarcinoma, distal cholangiocarcinoma, ampullary adenocarcinoma	OS/DFS	PubMed, Embase, WOS, Cochrane Library, and CNKI databases	To 28 October, 2024	—	15	2106	Low vs. high
Lv et al., 2020 [59]	Biliary tract cancer	OS	PubMed, Embase, WOS, and the Cochrane Library	To 7 December, 2019	Retrospective	7	1608	Low vs. high
Man et al., 2018 [47]	Hepatocellular carcinoma	OS/DFS	PubMed, Embase, and WOS	To 30 November, 2017	Retrospective	13	3738	Low vs. high
Pierce et al., 2024 [33]	Colorectal cancer; gastrointestinal; hepatocellular cancer;	OS/RFS/postoperative complications	PubMed, WOS, Google Scholar, and Embase	To 1 July, 2022	—	38	23,970	Low vs. high
Ren et al., 2022 [75]	Gastrointestinal stromal tumors	RFS	PubMed, WOS, CBM, Embase, and Cochrane	To 1 May, 2022	Retrospective	8	2462	Low vs. high
Sun et al., 2019 [50]	Colorectal cancer	OS	PubMed, Embase, and the WOS	To October 2018	Retrospective	15	6372	Low vs. high
Xue et al., 2019 [54]	Esophageal cancer	OS/CSS	PubMed, Embase, and Cochrane	To 3 December, 2018	Retrospective	11	3425	Low vs. high
Yang et al., 2016 (a) [85]	Gastric cancer	OS	PubMed, Embase, and WOS	To 30 November, 2015	Retrospective	10	3396	Low vs. high
Yang et al., 2016 (b) [84]	Colorectal cancer	OS/CSS	PubMed, Embase, and WOS	To 31 December, 2015	Retrospective cohort	11	3788	Low vs. high
Yu et al., 2024 [38]	Pancreatic neoplasms	OS	PubMed, Embase, WOS, Cochrane Library	To December 2023	Retrospective or prospective	27	6060	Low vs. high
Zeng et al., 2025 [42]	Advanced biliary tract cancers	OS/DFS	PubMed, Embase, and WOS	To April 2024	Retrospective	17	4645	Low vs. high
Zhang et al., 2023 [81]	Gastrointestinal cancer	OS/PFS/objective response rate/disease control rate	PubMed, the Cochrane Library, Embase, and Google Scholar	To 23 April, 2023	Retrospective and prospective	17	2883	High vs. low
Zhang et al., 2024 (a) [41]	Hepatocellular carcinoma	OS/RFS/DFS	Embase, PubMed, the Cochrane Library, WOS, and CNKI	To 3 April, 2024	Retrospective	19	9830	Low vs. high
Zhao et al., 2016 [48]	Gastric cancer, colorectal cancer, hepatocellular carcinoma, pancreatic cancer, esophageal carcinoma	OS/DFS	PubMed, ISI WOS	To 12 January, 2016	Retrospective	23	7384	Low vs. High
Zhao et al., 2022 [72]	Pancreatic cancer	OS/RFS	PubMed, Embase, WOS, and Cochrane Library databases	To 1 March, 2022	Retrospective	14	3385	Low vs. high
Respiratory system cancers								
Jiang et al., 2020 [62]	Small-cell lung cancer	OS/PFS	PubMed, Embase, and WOS	To 17 April, 2020	Retrospective	9	4164	Low vs. high
Li et al., 2018 (b) [45]	Lung cancer	OS	PubMed, Embase, and WOS	To 15 August, 2017	Retrospective	10	5085	Low vs. high
Shao et al., 2022 [74]	Lung cancer	OS/PFS	Embase, PubMed, Cochrane Library, American Society of Clinical Oncology, and European Society of Medical Oncology databases	To 2022	Retrospective	8	831	Low vs. high
Wang et al., 2018 [46]	Lung cancer	OS/DFS/RFS/PFS	PubMed, Embase, WOS, Wan Fang, and CNKI	To 1 April, 2018	Retrospective	21	—	Low vs. high
Wang et al., 2024 [43]	Lung cancer	OS/PFS	MEDLINE, CNKI, Embase, and WOS	To 20 August, 2023	Retrospective	22	2550	High vs. low
Xia et al., 2023 [80]	Advanced non–small-cell lung cancer	OS/PFS	PubMed, Embase, and Cochrane Library databases	To 13 August, 2022	Retrospective cohort	13	1119	Low vs. high
Yan et al., 2023 [11]	Advanced lung cancer	OS/PFS	PubMed, Embase, Cochrane Library, WOS, and Clinicaltrials.gov	To 1 March, 2023	Retrospective	14	1260	Low vs. high
Zhang et al., 2021 [68]	Lung cancer	PFS/OS	WOS, Embase, CENTRAL, Scopus, and MEDLINE databases	To April 2021	Retrospective cohort	16	4250	Low vs. high
Urinary system cancers								
Jiao et al., 2023 [82]	Bladder cancer	OS/RFS	PubMed, CENTRAL, Scopus, WOS, Embase, and Google Scholar	To 13 April, 2023	—	13	4413	Low vs. high
Kim et al., 2021 [64]	Renal cell carcinoma	OS/CSS/RFS/DFS	PubMed, Cochrane Central Search library, and Embase databases	To 1 May, 2020	Retrospective, observational, or case-control	9	5976	Low vs. high
Mao et al., 2021 [69]	Renal cell carcinoma	OS/PFS/DFS/RFS/poor cancer-specific survival	PubMed, WOS, Embase, Scopus, and Cochrane Library databases	To February 2021	Retrospective cohort	10	4908	Low vs. high
Meng et al., 2022 [73]	Upper tract urothelial carcinoma	OS/CSS/DSS/RF/PFS/DFS	PubMed, Embase, Scopus database, and Cochrane Library	To April 2022	Retrospective	6	2324	Low vs. high
Qi et al., 2018 [44]	Urinary cancers	OS/DFS/RFS/PFS	PubMed, Embase, and WOS	To November 2018	Retrospective	12	6561	Low vs. high
Sun et al., 2025 [37]	Bladder cancer	OS/RFS	PubMed, Embase, and the Cochrane Library	To April 2024	Prospective or retrospective	12	2951	Low vs. high
Tang et al., 2021 [63]	Renal cell carcinomas	OS/RFS	PubMed, WOS, Embase, and Cochrane Library	To 1 January, 2019	Retrospective	6	5479	Low vs. high
Zhao et al., 2019 [87]	Urologic cancer	OS	PubMed, Embase, and WOS	To 12 October, 2017	Retrospective	12	6786	Low vs. high
Reproductive system cancers								
Cao and Dong, 2024 [14]	Cervical cancer	OS/PFS	MEDLINE, Google Scholar, Science Direct, and Cochrane Central databases	To April 2023	Prospective and retrospective	10	2352	—
Li et al., 2024 [32]	Ovarian and endometrial cancer	OS/DFS/PFS	PubMed, Embase, WOS, and Cochrane Library databases	To 30 January, 2024	Retrospective and prospective cohort	28	9428	Low vs. high
Mao et al., 2024 [39]	Endometrial cancer	OS/inferior progression-free survival/DFS/RFS	PubMed, WOS, Embase, Cochrane Library, and CNKI databases	To 5 January, 2024	Prospective or retrospective	8	3164	Low vs. high
Niu and Yan, 2023 [13]	Cervical cancer	OS/PFS	Embase, PubMed, Cochrane Library, WOS, and CNKI	To 16 March, 2023	Retrospective	9	2508	Low vs. high
Tan and Chen, 2022 [71]	Ovarian cancer	OS/PFS/CSS	WOS, Embase, PubMed, CNKI, and WanFang Dataset	To 22 March, 2022	Retrospective and prospective observational	12	3190	High vs. low
Tobing et al., 2024 [36]	Prostate cancer	OS/PFS	Scopus, MEDLINE, CNKI, and Cochrane Library	To 9 December, 2023	Retrospective	13	2229	Low vs. high
Wang and Wang, 2019 [52]	Gynecological cancer	OS/PFS	PubMed, Embase, the Cochrane Library, WOS	To December 2018	Prospective and observational retrospective	9	2373	—
Zhang et al., 2024 (b) [40]	Endometrial cancer	OS/PFS	PubMed, Embase, WOS, Wanfang, and CNKI	To 25 April, 2024	Retrospective	10	3656	Low vs. high
Zheng et al., 2023 [79]	Prostate cancer	OS/PFS	PubMed, Embase, WOS, Cochrane Library (CENTRAL), and CNKI databases	To 1 March, 2023	Retrospective	10	1631	Low vs. high
Head and neck cancers								
Dai et al., 2023 [78]	Oral cancer	OS/CSS/DFS	PubMed, Embase, CNKI, Cochrane Library, and WOS	To 26 April, 2022	Retrospective	10	3130	Low vs. high
Luan et al., 2021 [65]	Head and neck cancer	OS/PFS/DSS/DFS/DMFS	PubMed, Embase, Cochrane Library	To June 2020	Retrospective	14	7815	Low vs. high
Shi et al., 2021 [66]	Head and neck neoplasms	OS/DMFS/PFS	PubMed, Embase, and WOS	To 30 November, 2020	Retrospective	10	3458	Low vs. high
Tang et al., 2020 [61]	Nasopharyngeal carcinoma	OS/PFS/locoregional failure-free survival/DMFS	Embase, PubMed, WOS, and the Cochrane Library	To April 2020	Retrospective cohort	8	3631	Low vs. high
Tu et al., 2020 [58]	Nasopharyngeal carcinoma	OS/DMFS	PubMed, WOS, Cochrane library, CNKI, and Wanfang database	To 25 July, 2020	Retrospective	10	1168	Low vs. high
Hematologic system cancers								
Luan et al., 2020 [60]	Diffuse large B-cell lymphoma	OS/PFS	CNKI, Wanfang, PubMed, Embase, the Cochrane Library, and WOS	To April 2020	—	7	1311	Low vs. high
Nervous system cancers								
Hung et al., 2023 [12]	Glioma	OS/PFS	MEDLINE, Embase, Google Scholar, and Cochrane Library	To 8 January, 2023	Retrospective	13	2712	High vs. low
Liu and Wang, 2020 [57]	Glioma	OS	PubMed, Embase, and Cochrane Library	To 31 December, 2019	Retrospective	11	2928	High vs. low
Breast cancers								
Prasetiyo et al., 2023 [83]	Breast cancer	OS/DFS	Cochrane Library, Scopus, Europe PMC, and MEDLINE databases	To 14 August, 2023	Retrospective	16	7457	High vs. low
Other cancers								
Bullock et al., 2020 [56]	Cancer	OS	Ovid MEDLINE, Embase, WOS, CINAHL, British Nursing Database, and Cochrane CENTRAL	To 8 December, 2018	Retrospective cohort	42	21032	Low vs. high
Li et al., 2022 (b) [70]	Advanced cancers	PFS/OS	PubMed, Embase (via OvidSP), and WOS	To 6 October, 2021	Retrospective observational	9	640	Low vs. high
Ni et al., 2022 [8]	Advanced-stage cancer	OS/PFS	PubMed, Embase, and ISI WOS	To June 2021	Retrospective and prospective	12	1359	Low vs. high
Sun et al., 2014 [86]	Cancer	OS/CSS	PubMed and ISI WOS	To 20 March, 2014	Retrospective observational	14	3414	Low vs. high
Xu et al. 2023 [9]	Cancer	OS/PFS/objective response rate/disease control rate/rate of adverse events	PubMed, Embase, and Cochrane Library databases	To July 2022	Retrospective	23	2386	Low vs. high

Abbreviations: CBM, The Chinese Biomedical Database; CENTRAL, Cochrane Central Register of Controlled Trials; CINAHL, Cumulative Index to Nursing and Allied Health Literature; CNKI, China National Knowledge Infrastructure; CSS, cancer-specific survival; DFS, disease-free survival; DMFS, distant metastasis-free survival; DSS, Disease-Specific Survival; ISI WOS, Web of Science launched by the Institute for Scientific Information; OS, overall survival; PFS, progression-free survival; RFS, recurrence-free survival.

### Methodological quality of included meta-analyses

The AMSTAR scores for the 64 included systematic reviews and meta-analyses ranged from 6 to 10, with a median score of 8. Of these, 44 (68.8%) were rated as high quality, and 20 (31.2%) as moderate quality (Supplementary Figure 1). During the AMSTAR assessment, the primary cause for the reduction in score was the absence of a list of including and excluding studies.

### Certainty of evidence

The application of the GRADE framework indicated that the evidence certainty was of high certainty for 121 quantitative syntheses, moderate certainty for 71 quantitative syntheses, low certainty for 52 quantitative syntheses, and very low certainty for 21 quantitative syntheses. The main reason for downgrading the confidence in findings was inconsistency across primary studies (*n =* 131), followed by publication bias (*n =* 77), imprecision (*n =* 10), and risk of bias. Furthermore, a large effect size was the primary reason for upgrading the certainty rating (*n =* 74) (Supplementary Table 5).

### Summary of the quantitative syntheses between PNI and the prognosis of different cancers

#### Digestive system cancers

This analysis synthesizes evidence from 80 quantitative syntheses investigating the association between the PNI and the prognosis of digestive system tumors. The included systematic reviews and meta-analyses covered various malignancies, including gastric cancer, colorectal cancer, hepatocellular carcinoma, pancreatic cancer, esophageal cancer, and biliary tract cancer. Seventy-three meta-analyses were statistically significant, and 49 used OS as the primary outcome, whereas others assessed outcomes such as RFS, PFS, and postoperative complications. The certainty of evidence for these meta-analyses varied, ranging from low to high: 31 were rated as high certainty, 30 as moderate, 14 as low, and 5 as very low. One analysis by Li et al. [10], which provided high-certainty evidence, included 15 primary studies on gastric cancer and demonstrated that a lower PNI was significantly associated with poorer OS compared with a higher PNI, with a pooled HR of 1.81 (95% CI: 1.56, 2.09).

#### Respiratory system cancers

This analysis synthesized evidence from 21 quantitative syntheses investigating the association between the PNI and the prognosis of respiratory system tumors. The malignancies examined included lung cancer, with specific subgroup analyses focusing on non–small-cell lung cancer, small-cell lung cancer, and advanced lung cancer. Fifteen meta-analyses were statistically significant, and 12 used OS as the primary endpoint, whereas the remaining evaluated PFS, DFS, and RFS. The certainty of evidence ranged from low to high across these meta-analyses, with 5 rated as high certainty, 3 as moderate certainty, 5 as low certainty, and 8 as very low certainty. One analysis by Yan et al. [11], which provided high-certainty evidence from 13 primary studies on advanced lung cancer, demonstrated that a lower PNI was significantly associated with inferior OS compared with a higher PNI, with a pool HR of 2.56 (95% CI: 1.86, 3.54).

#### Urinary system cancers

This analysis synthesizes evidence from 39 quantitative syntheses investigating the association between the PNI and the prognosis of urinary system tumors, including bladder cancer, renal cell carcinoma, upper tract urothelial carcinoma, and other urologic malignancies. Thirty-eight meta-analyses were statistically significant, and 19 used OS as the primary endpoint, whereas the remaining assessed RFS, PFS, DFS, and CSS. The certainty of evidence was predominantly moderate to high, with 28 quantitative syntheses rated as high certainty, 10 as moderate certainty, and 1 as low certainty. An analysis by Sun et al. [37], which provided high-certainty evidence from 10 primary studies on bladder cancer, demonstrated that a lower PNI was significantly associated with inferior OS compared with a higher PNI, with a pooled HR of 1.80 (95% CI: 1.54, 2.10).

#### Reproductive system cancers

This analysis synthesizes evidence from 65 quantitative syntheses investigating the association between the PNI and the prognosis of reproductive system tumors, including endometrial, cervical, ovarian, and prostate cancers, among other reproductive malignancies. Forty-three meta-analyses were statistically significant, and 40 used OS as the primary endpoint, whereas the remainder assessed outcomes such as PFS and DFS. The certainty of evidence varied across meta-analyses: 16 were rated as high certainty, 15 as moderate, 26 as low, and 8 as low. One analysis by Niu et al. [13], which included 8 primary studies on cervical cancer, provided high-certainty evidence. This analysis demonstrated that a lower PNI was significantly associated with poorer OS compared with a higher PNI, with a pooled HR of 2.98 (95% CI: 2.22, 3.99).

#### Head and neck cancers

This analysis synthesizes evidence from 22 quantitative syntheses investigating the association between the PNI and the prognosis of head and neck tumors, including oral cancer, nasopharyngeal carcinoma, and other head and neck neoplasms. Twenty-two meta-analyses were statistically significant, and 8 used OS as the primary endpoint, whereas the remainder assessed PFS, distant metastasis-free survival, DFS, and RFS. The certainty of evidence was predominantly low to high, with 15 quantitative syntheses rated as high certainty, 4 as moderate certainty, and 3 as low certainty. An analysis by Luan et al. [65], which provided high-certainty evidence from 6 primary studies on head and neck cancer, demonstrated that a lower PNI was significantly associated with inferior OS compared with a higher PNI, with a pooled HR of 2.04 (95% CI: 1.74, 2.38).

#### Hematologic cancers

This synthesis evaluated evidence from 2 meta-analyses examining the association between the PNI and survival outcomes in hematologic malignancies, with specific focus on diffuse large B-cell lymphoma. Both demonstrated high-certainty evidence according to GRADE criteria.

#### Nervous system cancers

The presented analysis summarizes 8 meta-analyses examining the association between the PNI and the prognosis of nervous system tumors. All included systematic reviews and meta-analyses specifically involved people with glioma. The certainty of evidence for these meta-analyses was rated as moderate to high, with 5 quantitative syntheses classified as high certainty, 1 as moderate certainty, and 2 as low certainty. One quantitative synthesis providing high-certainty evidence was reported by Hung et al. [12]. This meta-analysis, which incorporated 13 primary studies, demonstrated that a higher PNI was significantly associated with improved OS compared with a lower PNI, with an HR of 0.61 (95% CI: 0.52, 0.72).

#### Breast cancers

This analysis synthesizes evidence from 2 quantitative syntheses examining the association between the PNI and survival outcomes in breast cancer. The certainty of evidence varied between the 2 meta-analyses: 1 demonstrated high certainty for OS [83], whereas another showed low certainty for DFS [83].

#### Other cancers

This analysis synthesizes evidence from 26 quantitative syntheses examining the association between the PNI and cancer prognosis across various malignancies, including both advanced-stage and general cancer populations. Twenty-four quantitative syntheses were statistically significant. The evaluated outcomes encompassed multiple endpoints, with 14 meta-analyses focusing on OS as the primary measure, whereas others assessed PFS, disease control rate, and objective response rate. The certainty of evidence was predominantly moderate to high, with 18 quantitative syntheses rated as high certainty and 8 as moderate certainty. One analysis by Xu et al. [9], which provided high-certainty evidence from 19 primary studies, demonstrated that a lower PNI was significantly associated with poorer PFS compared with a higher PNI, with a pooled HR of 1.75 (95% CI: 1.54, 1.99).

### Sensitivity analyses

Sensitivity analyses confirmed the overall robustness of the findings, despite minor variations (Supplementary Tables 6 and 7). Exclusion of studies with small sample sizes (primary studies with sample sizes falling below the 25th percentile of the distribution within that specific meta-analysis) yielded a summary effect size that remained largely consistent. However, this exclusion increased heterogeneity in 2 meta-analyses, calculated by a rise in *I*^*2*^ from 43% to 54.8% [72] and from 49.3% to 56.2% [10], leading to a downgrade in the GRADE certainty of evidence from high to moderate for these quantitative syntheses. In the sensitivity analysis excluding primary studies with a high risk of bias, effect size was highly consistent with the primary results. For 4 meta-analyses [8,11,43,86], no primary studies were rated as having a high risk of bias; thus, it is not suitable to conduct a sensitivity analysis. All other GRADE evidence certainty ratings remained unchanged.

## Discussion

### Main findings

**FIGURE 2:**
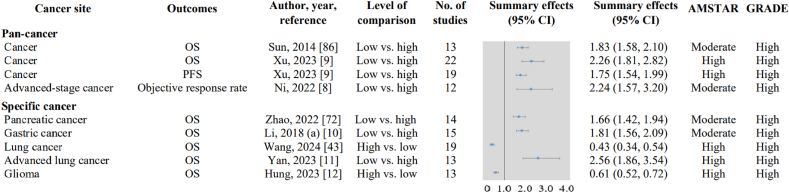
Summary random-effects estimate from 9 associations of prognostic nutritional index and cancer prognostic outcomes (primary studies >10). AMSTAR, A Measurement Tool to Assess Systematic Reviews; CI, confidence interval; GRADE, Grading of Recommendations, Assessment, Development and Evaluation; OS, overall survival; PFS, progression-free survival.

This UR evaluated the certainty of evidence linking the PNI with cancer prognostic outcomes using the GRADE framework. Among 265 quantitative syntheses assessed, 9 were supported by high-certainty evidence from >10 studies (Figure 2). A higher PNI was consistently associated with improved OS in people with gastric, pancreatic, and lung cancers, as well as across multiple other malignancies. Similarly, elevated PNI correlated with prolonged PFS and improved objective response rates in advanced-stage cancers. These findings robustly affirm the prognostic value of PNI across diverse cancer types and clinical endpoints.

Our research has demonstrated that patients with cancer with elevated preoperative PNI scores exhibit longer OS durations compared with those with lower scores, aligning with previous findings [38]. PNI has been validated as a standalone predictor for OS and postoperative complications, yet it does not correlate with CSS rates [86]. Our study has shown that the PNI is significantly associated with the prognosis of people with gastric cancer and esophageal cancer [89], potentially attributable to the direct influence of nutritional status on upper gastrointestinal malignancies [90]. Our findings offer profound insights into the prognostic value of PNI across diverse cancer types. However, to comprehensively grasp its clinical implications, further investigation is warranted to elucidate the relationship between PNI and the prognosis of patients with specific cancers.

Our study demonstrated that patients with gastric cancer with higher preoperative PNI scores exhibited significantly longer OS compared with those with lower PNI scores, aligning with the findings of previous studies [10,48,85]. The association between higher PNI and improved OS can be attributed to several factors. First, PNI reflects both nutritional and immunological status, with serum albumin and lymphocyte count serving as key indicators. Higher serum albumin concentrations correlate with better nutritional status and reduced systemic inflammation, whereas robust lymphocyte counts enhance the immune system’s ability to combat tumor progression [91,92]. Second, people with higher PNI scores often present with more favorable clinicopathological features, such as earlier tumor stages, less lymphatic or vascular invasion, and lower rates of postoperative complications, all of which contribute to prolonged survival [85]. Conversely, malnutrition and immune impairment associated with low PNI may delay or preclude adjuvant therapies, further exacerbating poor outcomes [10,48]. Previous studies have shown that low serum albumin concentrations in people with gastric cancer are associated with poor prognosis [93,94]. However, the causal relationship between PNI and tumor progression remains unclear, warranting further investigation. In addition, the optimal PNI cutoff value for prognostic stratification requires validation, because current studies report varying thresholds (40–49.7) [85,95].

Present research indicated that patients with pancreatic cancer with elevated preoperative PNI scores exhibit significantly longer OS compared with their counterparts with lower scores, a finding that corroborates results from previous studies [34,38,49,72]. Hypoalbuminemia, a component of low PNI, is indicative of malnutrition and cachexia, conditions prevalent in pancreatic cancer due to pancreatic exocrine insufficiency and systemic inflammation [96], which adversely affect patient outcomes. Simultaneously, lymphocytes play a pivotal role in antitumor immunity, and their depletion, often observed in pancreatic cancer, correlates with disease progression and poor prognosis [97]. Thus, a higher PNI score signifies better nutritional and immunological reserves, enabling patients to tolerate aggressive treatment [98]. These findings underscore the potential of PNI as a reliable, cost-effective prognostic tool in pancreatic cancer management, guiding personalized treatment strategies and improving patient survival.

Our research indicates that patients with lung cancer with elevated preoperative PNI scores exhibit prolonged OS when compared with those with diminished scores, a finding that aligns with preceding studies [45,46]. Prior study has unveiled a correlation between PNI and both the gender and histological subtype of patients with lung cancer [46]. Notably, a low PNI state predominates among male patients and those diagnosed with nonadenocarcinoma, potentially attributable to the distinct biological traits and treatment responses inherent to varying genders and histological types [45,99]. Specifically concerning non–small-cell lung cancer, previous meta-analysis has corroborated that a low PNI is indicative of a reduced RFS [46]. This association has been substantiated through subgroup analyses encompassing diverse histological types, TNM stages, geographical regions of study, and sample sizes [45]. In the context of small-cell lung cancer, it was similarly observed that a low PNI correlated with a shorter OS; however, the relationship between PNI and PFS necessitates further empirical validation [62]. Moreover, many studies elucidate that for patients with lung cancer undergoing treatment with immune checkpoint inhibitors, a low PNI is predictive of a less favorable prognosis [11]. People with a low PNI might encounter an escalated risk of immune-related toxicities during therapy, potentially due to the impaired immune function precipitated by low lymphocyte counts [43]. The pivotal role of inflammation and the immune system in the onset and progression of cancer further underscores the rationale for employing PNI as a prognostic indicator.

Our research has revealed that patients with glioma with elevated preoperative PNI scores exhibit significantly longer OS compared with those with lower scores. The underlying mechanism for this observation may be attributed to the fact that low albumin concentrations, a component of PNI, are closely associated with malnutrition and inflammation, both recognized as contributing factors to cancer initiation and progression [100]. Malnutrition often manifests as weight loss, whereas inflammation adversely affects cancer prognosis by promoting tumor cell proliferation, migration, and immune evasion [101]. Furthermore, the prognostic benefit of PNI may also arise from its capacity to reflect the patient’s lymphocyte and globulin concentrations. Lymphocytes play a crucial role in antitumor immunity, whereas globulins, under certain circumstances, may facilitate tumor progression [102]. Subgroup analyses, stratified by race, sample size, and the source of effect size (univariate compared with multivariate), consistently corroborated the prognostic value of PNI [12]. The utility of PNI as a prognostic indicator is supported by multiple studies, demonstrating a stronger correlation with glioma prognosis compared with other individual nutritional or inflammatory markers, such as the neutrophil-to-lymphocyte ratio, lymphocyte-to-monocyte ratio, and platelet-to-lymphocyte ratio [12]. Traditional prognostic factors for glioma encompass tumor grade, location, and biomarker genes [12]. In contrast, PNI offers a noninvasive, cost-effective, and straightforward prognostic tool that can supplement existing prognostic information and facilitate the development of more personalized treatment strategies.

### Strengths and limitations

This UR represented the first comprehensive synthesis of evidence on the prognostic utility of the PNI across diverse cancer types. By aggregating results from multiple meta-analyses of observational studies, it offers a broad overview of PNI’s role in predicting clinical outcomes. The GRADE framework was applied to evaluate the confidence in the evidence, enhancing transparency and interpretability. To ensure consistency across studies, all effect estimates were recalculated using random-effects models, improving the comparability of results. Methodological quality was assessed using the AMSTAR tool, which revealed that 66.7% of the included meta-analyses met high-quality standards. However, a methodological study indicated that the majority of systematic reviews were of critically low quality (80.1%) [103], a finding that starkly contrasted with our results. This discrepancy arose from the use of different tools for assessing methodological quality; our study applied the relatively lenient AMSTAR tool. Moreover, the key advantages of this UR lie in its systematic integration and rigorous evaluation of the wide variations in PNI cutoff values across the entire field. It emphasizes the necessity for future research to standardize the determination of cutoff values. In addition, the large sample size and broad coverage of cancer types—including digestive, respiratory, urinary, and other malignancies—allow for site-specific subgroup analyses, providing more nuanced insights into PNI’s prognostic value.

Several potential limitations warrant consideration in our study. First, this UR is based exclusively on meta-analyses derived from observational studies, which are inherently susceptible to biases such as selection and confounding at the primary study level. Nevertheless, RCTs examining the association between the PNI and cancer prognostic outcomes were scarce. In prognosis research, observational studies were more pertinent to our focus due to their benefits, including larger sample sizes, an adequate number of studies, and extended follow-up periods [104]. Second, the derivation of the UR was predicated upon previously published systematic reviews that incorporated meta-analyses. This approach may inadvertently lead to the exclusion of certain primary studies that were not captured within the scope of those reviews. Nevertheless, to mitigate this potential limitation and ensure the comprehensiveness of the included studies, we undertook a meticulous examination of the reference lists from the studies incorporated in the systematic reviews. Third, we excluded systematic reviews that performed only qualitative syntheses or lacked detailed data for individual studies. This exclusion raised the possibility of misestimating the findings. However, we synthesized results from these studies to ensure a comprehensive consideration of all relevant research, as outlined in Supplementary Table 8. Fourth, among the total of 265 meta-analyses conducted in this study, 67 showed high heterogeneity, and 64 exhibited extremely high heterogeneity. The sources of heterogeneity might include differences in factors. The differences in clinical characteristics such as cancer stage and treatment modalities (e.g., surgery alone compared with combined regimens), and methodological inconsistencies including follow-up duration and covariate adjustment in multivariate analyses. These sources of variability warrant careful consideration when interpreting pooled estimates and underscore the need for greater standardization in future prognostic research. Moreover, the substantial heterogeneity in PNI cutoff values, which ranged from 31.1 to 57.0, along with the diverse methods used to determine these thresholds (such as receiver operating characteristic curves, medians, or study-specific quartiles), precluded meaningful stratified analyses in this UR. Future studies should aim to harmonize PNI definitions or conduct sensitivity analyses by clinically relevant cutoff ranges to enhance result interpretability and applicability. In addition, several meta-analyses within our UR included <10 primary studies, which may limit the statistical power of publication bias assessments, such as Begg’s or Egger’s tests [105]. Therefore, interpretations of publication bias should be made with caution.

In conclusion, this study establishes the PNI as a significant prognostic value across diverse cancer types. This finding underscores its potential utility in guiding and enhancing tertiary prevention strategies for patients with cancer. A higher PNI demonstrates a consistent association with improved OS in major malignancies, including gastric, pancreatic, gliomas, and lung cancers. However, significant heterogeneity in PNI threshold definitions across studies remains a critical challenge. Future, large-scale, multicenter, prospective studies are imperative to establish population-specific PNI cutoffs. This is an essential step for translating this robust evidence into consistent and effective clinical practice.

## Author contributions

The authors’ responsibilities were as follows – Z-TL, M-LS, F-HL: were joint first authors; Z-TL, M-LS, T-TG, T-GL, Q-JW: conceived the study and contributed to the design; Z-TL, M-LS, F-HL, YL, R-HB, X-FJ, JL, ZL, QC, QF, Z-FF, X-XJ, D-RL, ML, JM, YW, Y-LX, DY, MY, RY, X-LB: collected, cleaned, and analyzed the data; Z-TL, M-LS, F-HL, YL, YYang, YYu, NZ, QC, Z-FF, D-RL, ML, JY, T-TG, T-GL, Q-JW: drafted the article and revised it critically for important intellectual content; T-TG, T-GL, Q-JW: agreed to be accountable for all aspects of the work; and all authors: read and approved the final manuscript.

## Declaration of generative AI and AI-assisted technologies in the writing process

The authors declare that no generative AI or AI-assisted technologies were used in the writing of this manuscript.

## Data availability

The data supporting the conclusions of this UR can be directed to the corresponding author.

## Funding

This work was supported by the Natural Science Foundation of China (No. 82373674 to Q-JW), Liaoning Province Educational Science Planning Project (No. JG22DB707 to Q-JW), Liaoning Province Science and Technology Plan (No. 2023JH2/20200019 to Q-JW), and Scientific Research Project of Education Department of Liaoning Province (No. LJKMZ20221137 to T-TG).

## Conflict of interest

The authors report no conflicts of interest.
