# Supplementary material for: Prognostic Nutritional Index and Cancer Prognostic Outcomes: An Umbrella Review of Systematic Reviews and Meta-analyses of Observational Studies
Source: Adv Nutr. 2026 Apr 17;17(6):100641. doi: 10.1016/j.advnut.2026.100641 (PMC13195311; doi:10.1016/j.advnut.2026.100641)
Supplement: multimedia component 1 [file mmc1.docx]

**Prognostic nutritional index and cancer prognostic outcomes: an umbrella review of systematic reviews and meta-analyses of observational studies**

Zhi-Tong Li et al.

**Supplementary materials**

**Supplementary table 1.** Search strategy from database inception to February 28, 2025

**Supplementary table 2.** Excluded studies with reasons from the search for meta-analyses during the full-text screening

**Supplementary table 3.** The summary of the cut-off values of PNI for included studies

**Supplementary table 4.** Summary of associations of PNI with cancer prognostic outcome

**Supplementary table 5.** The results of GRADE assessment of the evidence certainty of PNI with cancer prognostic outcomes

**Supplementary table 6.** Summary of sensitivity analysis after excluding small sample size (25th percentile) studies significant associations in high-certainty evidence from more than ten studies

**Supplementary table 7.** Summary of sensitivity analysis after excluding high risk of bias studies significant associations in high-certainty evidence with more than ten studies

**Supplementary table 8.** The summary results of meta-analyses excluded due to without quantitative synthesis

**Supplementary figure 1.** Methodological quality assessment of the included articles with AMSTAR

**Supplementary table 1. Search strategy from database inception to February 28, 2025**

| **Database** | **Search term** | **Results** |
| --- | --- | --- |
| PubMed | #1 (PNI) OR (prognostic nutritional index)  #2 (((meta-analysis) OR (systematic review)) OR (systematic overview)) OR (meta-analyses)  #3 (((((cancer) OR (carcinoma)) OR (tumour)) OR (neoplasm)) OR (tumor)) OR (neoplasms)  #4 (((((survival) OR (recurrence)) OR (prognosis)) OR (progress)) OR (outcome)) OR (predict)  #5 #1 AND #2 AND #3 AND #4 | 767 |
| Web of Science | #1 (TS=(PNI)) OR TS=(prognostic nutritional index)  #2 (((TS=(meta-analysis) OR TS=(systematic review)) OR TS=(systematic overview)) OR TS=(meta-analyses)  #3 (((((TS=(cancer) OR TS=(carcinoma)) OR TS=(tumour)) OR TS=(neoplasm)) OR TS=(tumor)) OR TS=(neoplasms)  #4 (((((TS=(survival) OR TS=(recurrence)) OR TS=(prognosis)) OR TS=(progress)) OR TS=(outcome)) OR TS=(predict)  #5 #1 AND #2 AND #3 AND #4 | 159 |
| Embase | #1 ‘PNI’  #2 ‘prognostic nutritional index’  #3 #1 OR #2  #4 ‘meta-analysis’  #5 ‘systematic review’  #6 ‘systematic overview’  #7 ‘meta-analyses’  #8 #4 OR #5 OR #6 OR #7  #9 ‘cancer’  #10 ‘carcinoma’  #11 ‘tumour’  #12 ‘neoplasm’  #13 ‘tumor’  #14 ‘neoplasms’  #15 #9 OR #10 OR #11 OR #12 OR #13 OR #14  #16 ‘survival’  #17 ‘recurrence’  #18 ‘prognosis’  #19 ‘progress’  #20 ‘outcome’  #21 ‘predict’  #22 #16 OR #17 OR #18 OR #19 OR #20 OR #21  #23 #3 AND #8 AND #15 AND #22 | 181 |
| Cochrane Database of Systematic Review | #1 (PNI) OR (prognostic nutritional index)  #2 (meta-analysis) OR (systematic review) OR (systematic overview) OR (meta-analyses)  #3 (cancer) OR (carcinoma) OR (tumour) OR (neoplasm) AND (tumor)  #4 (neoplasms)  #5 #3 OR #4  #6 (survival) OR (recurrence) OR (prognosis) OR (progress) OR (outcome)  #7 (predict)  #8 #6 OR #7  #9 #1 AND #2 AND #5 AND #8 | 109 |
| **Total** |  | **1216** |

**Supplementary table 2. Excluded studies with reasons from the search for meta-analyses during the full-text screening**

| **Reasons for exclusion** | **References** |
| --- | --- |
| Systematic review without meta-analysis (n=8) | 1. Grimes N, Tyson M, Hannan C, Mulholland C. A Systematic Review of the Prognostic Role of Hematologic Scoring Systems in Patients with Renal Cell Carcinoma Undergoing Nephrectomy With Curative Intent. Clin Genitourin Cancer 2016; 14(4): 271-6. 2. Fiflis S, Christodoulidis G, Papakonstantinou M, et al. Prognostic nutritional index in predicting survival of patients with gastric or gastroesophageal junction adenocarcinoma: A systematic review. World J Gastrointest Oncol 2024; 16(2): 514-26. 3. Yan L, Nakamura T, Casadei-Gardini A, Bruixola G, Huang YL, Hu ZD. Long-term and short-term prognostic value of the prognostic nutritional index in cancer: a narrative review. Ann Transl Med 2021; 9(21): 1630. 4. Teja M, Garrido MI, Ocanto A, Counago F. Prognostic impact of inflammatory and nutritional biomarkers in pancreatic cancer. World J Clin Oncol 2025; 16(1): 101191. 5. Morelli I, Greto D, Visani L, et al. Integrating nutritional status and hematological biomarkers for enhanced prognosis prediction in glioma patients: A systematic review. Clin Nutr ESPEN 2025; 66: 269-80. 6. Laszkiewicz J, Krajewski W, Sojka A, et al. Blood-, Tissue- and Urine-Based Prognostic Biomarkers of Upper Tract Urothelial Carcinoma. Diagnostics (Basel) 2024; 14(17). 7. Feng YW, Wang HY, Lin Q. Can the preoperative prognostic nutritional index be used as a postoperative predictor of gastric or gastroesophageal junction adenocarcinoma? World J Gastrointest Oncol 2024; 16(7): 2877-80. 8. Li CJ, Lee PC, Huang KW, et al. Postoperative prognostic nutrition index predicts survival in patients with small bowel adenocarcinoma after surgical resection. Journal of the Chinese Medical Association: JCMA 2024; 87(9): 819-27. |
| Missing data (n=7) | 1. Liu J, Jiang S, Yang X, Li X, Wang N. The Significant Value of Preoperative Prognostic Nutritional Index for Survival in Pancreatic Cancers: A Meta-analysis. Pancreas 2018; 47(7): 793-9. 2. Yang J, Li H, Li L, Lv J. Prognostic Role of Pretreatment Prognostic Nutritional Index in Advanced Lung Cancer Patients Receiving First-Line Immunotherapy: A Meta-Analysis. Cureus 2024; 16(1): e52720. 3. Xue S, Zhao H, Zhang K, Zhang H, Wang W. Prognostic and Clinicopathological Correlations of Pretreatment Prognostic Nutritional Index in Renal Cell Carcinoma: A Meta-Analysis. Urol Int 2022; 106(6): 567-80. 4. Hu G, Ding Q, Zhong K, Wang S, Wang S, Huang L. Low pretreatment prognostic nutritional index predicts poor survival in breast cancer patients: A meta-analysis. PLoS One 2023; 18(1): e0280669. 5. Xu J, Lin Y, Yang J, Xing Y, Xing X. Pretreatment systemic immune-inflammation index and lymphocyte-to-monocyte ratio as prognostic factors in oral cavity cancer: A meta-analysis. Medicine (Baltimore) 2024; 103(44): e40182. 6. de Castro e Borges F, Khajeh E, Nikbakhsh R, et al. Preoperative Inflammatory Scores Do Not Accurately Predict Early Recurrence of Pancreatic Ductal Adenocarcinoma After Resection: A Systematic Review and Meta-Analysis. Cancer Medicine 2024; 13(20): e70352 7. Li S, Zhang H, He J, Li S. Relationship Between Preoperative Nutritional Indicators and Postoperative Complications in Patients with Oesophageal Cancer: A Meta-Analysis. Nutr Cancer 2024; 76(7): 563-72. |
| Absence of cancer prognostic outcome measures (n=1) | 1. Shim SR, Kim SI, Kim SJ, Cho DS. Prognostic nutritional index as a prognostic factor for renal cell carcinoma: A systematic review and meta-analysis. PLoS One 2022; 17(8): e0271821. |
| Small number of studies with same exposure and same outcome (n=8) | 1. Wang SH, Zhai ST, Lin H. Role of Prognostic Nutritional Index in patients with gastric cancer: a meta-analysis. Minerva Med 2016; 107(5): 322-7. 2. Peng J, Li X, Huang M, et al. Prognostic value of prognostic nutritional index score and controlling nutritional status score in patients with glioblastoma: A comprehensive meta-analysis. Front Oncol 2023; 13: 1117764. 3. Hu Y, Shen J, Liu R, et al. Prognostic value of pretreatment prognostic nutritional index in non-small cell lung cancer: A systematic review and meta-analysis. Int J Biol Markers 2018; 33(4): 372-8. 4. Hao J, Chen C, Wan F, et al. Prognostic Value of Pre-Treatment Prognostic Nutritional Index in Esophageal Cancer: A Systematic Review and Meta-Analysis. Front Oncol 2020; 10: 797. 5. Xiong SC, Hu X, Lia T, Wang YH, Li X. Prognostic Significance of Prognostic Nutritional Index in Patients with Renal Cell Carcinoma: A Meta-Analysis. Nutr Cancer 2022; 74(3): 860-8. 6. Gao QL, Shi JG, Huang YD. Prognostic Significance of Pretreatment Prognostic Nutritional Index (PNI) in Patients with Nasopharyngeal Carcinoma: A Meta-Analysis. Nutr Cancer 2021; 73(9): 1657-67. 7. Dai Y, Liu M, Lei L, Lu S. Prognostic significance of preoperative prognostic nutritional index in ovarian cancer: A systematic review and meta-analysis. Medicine (Baltimore) 2020; 99(38): e21840. 8. Peng P, Chen L, Shen Q, Xu Z, Ding X. Prognostic Nutritional Index (PNI) and Controlling Nutritional Status (CONUT) score for predicting outcomes of breast cancer: A systematic review and meta-analysis. Pak J Med Sci 2023; 39(5): 1535-41 |

**Supplementary table 3. The summary of the cut-off values of PNI for included studies**

| **Author, year, ref** | **Cut-off values of PNI** |
| --- | --- |
| Bullock, 2020 ^1^ | It varied from 40 to 49.6 (Table 1). |
| Cao, 2024 ^2^ | It ranged between 45‑55. |
| Dai, 2023 ^3^ | Subgroup: <=49 (N=5); >49 (N=4). |
| Deng, 2024 ^4^ | Subgroup: >45 (N=14); <=45 (N=15). |
| Fan, 2019 ^5^ | Range: 45 to 52. |
| Hou, 2024 ^6^ | Subgroup: >=40 (N=4); <40 (N=2). |
| Hung, 2023 ^7^ | Range: 43.38 to 52.55 |
| Jiang, 2020 ^8^ | Median: 48.5 (range, 37.5–53.9). |
| Jiang, 2021 ^9^ | No available |
| Jiao, 2023 ^10^ | Subgroup: >=50 (N=3); <50 (N=3). |
| Kang, 2022 ^11^ | Range: 37.5 to 51.3, with a median value of 47.46. |
| Kim, 2021 ^12^ | Range: 41 to 51. |
| Li, 2018 (a) ^13^ | Range: 40 to 52. 32% of the studies (8 of 25) having a cutoff value set at 45. |
| Li, 2018 (b) ^14^ | Range: 46.24 to 52.48. |
| Li, 2019 (a) ^15^ | Range: 35 to 49.5. |
| Li, 2019 (b) ^16^ | Subgroup: >45 (N=5); <=45 (N=3). |
| Li, 2022 (a) ^17^ | Range: 40.7 to 51.3. |
| Li, 2022 (b) ^18^ | Range:31.1 to 48. |
| Li, 2024 ^19^ | Range: 38 to 52.83. |
| Liao, 2019 ^20^ | Subgroup: >=46 (N=6); <46 (N=5). |
| Liu, 2020 ^21^ | Range: 44.4 to 48. |
| Liu, 2025 ^22^ | Range: 36.90 to 48.85. |
| Luan, 2020 ^23^ | Range: 40 to 45. |
| Luan, 2021 ^24^ | Range: 40 to 56.93. |
| Lv, 2020 ^25^ | Range: 36.7 to 48.25. |
| Man, 2018 ^26^ | Median: 45 (Range: 41-52). |
| Mao, 2021 ^27^ | Range: 38.5 to 51.62, with a median value of 46.31. |
| Mao, 2024 ^28^ | Subgroup: <50 (N=3); >=50 (N=5). |
| Meng, 2022 ^29^ | Range: 45 to 50.5. |
| Ni, 2022 ^30^ | Range: 31.1 to 46.05. The value being 40 and 45 in 3 studies, respectively. |
| Niu, 2023 ^31^ | Median: 48.82 (range: 45 to 52.68). |
| Pierce, 2024 ^32^ | No available |
| Prasetiyo, 2023 ^33^ | Range: 48.7 to 55. |
| Qi, 2018 ^34^ | Range: 44.7 to 52.57. |
| Ren, 2022 ^35^ | Range: 42.6 to 51.3. |
| Shao, 2022 ^36^ | Range: 40.35 to 48. |
| Shi, 2021 ^37^ | Range: 42.7 to 55. It was determined by 3 methods: ROC curve, Cutoff Finder (a web application), and median. |
| Sun, 2014 ^38^ | Range: 40 to 49.7. It being 45 in 6 studies. |
| Sun, 2019 ^39^ | Range: 35 to 49.22; three studies (of 10) set 45 as the cutoff value and two studies (of 10) used 45.5. |
| Sun, 2025 ^40^ | Range: 40.00 to 52.57. |
| Tan, 2022 ^41^ | Range: 42.9 to 50.4 |
| Tang, 2020 ^42^ | Range: 45.58 to 55 (for OS), 45.45 to 55 (for PFS), and 45.58 to 55 (for DMFS). |
| Tang, 2021 ^43^ | Range: 41 to 51. |
| Tobing, 2024 ^44^ | Subgroup: <=50 (N=6); >50 (N=3). |
| Tu, 2020 ^45^ | Median: 51 |
| Wang, 2018 ^46^ | Subgroup: 45-45.2 (N=3); 45.95-47.9 (N=3); 49.5-50 (N=3); > 50 (N=4). |
| Wang, 2019 ^47^ | Range: 45 to 50.38. |
| Wang, 2024 ^48^ | Range: 40 to 50.5. |
| Xia, 2023 ^49^ | Range: 40 to 50. |
| Xu, 2023 ^50^ | Subgroup: <45 (N=10); >45 (N=12). |
| Xue, 2019 ^51^ | Range: 42 to 57. |
| Yan, 2023 ^52^ | Median: 45.05. |
| Yang, 2016 (a) ^53^ | Subgroup: (<=45.5 vs. >45.5, median of 45.5). |
| Yang, 2016 (b) ^54^ | Range: 40.0 to 45.5 (median: 45.0). |
| Yu, 2024 ^55^ | Subgroup: <=45 (N=6); >45 (N=9). |
| Zeng, 2025 ^56^ | Range: 36.7 to 48.0. |
| Zhang, 2021 ^57^ | Range: 40 to 53.85. |
| Zhang, 2023 ^58^ | Subgroup: >45 (N=2); 40-45 (N=7); <40 (N=2). |
| Zhang, 2024 (a) ^59^ | Range: 44.35 to 53.95. |
| Zhang, 2024 (b) ^60^ | Range: 38 to 52.8. |
| Zhao, 2016 ^61^ | Range: 40 to 49.7. 43.5% of the studies (10 of 23) having a cut-off value set at 45. |
| Zhao, 2019 ^62^ | Subgroup: >50 (N=3); <50 (N=6). |
| Zhao, 2022 ^63^ | Range: 36 to 53.10. |
| Zheng, 2023 ^64^ | Subgroup: <=49.2 (N=3); >49.2 (N=4). |

**Supplementary table 4. Summary of associations of prognostic nutritional index with cancer prognostic outcome.**

| **Author, year, ref** | **Cancer site** | **Outcomes** | **Level of comparison** | **Analysis Type** | **No. of studies** | **Effect metric** | **Summary effects (95% CI)** | **Random *P* value** | ***I^2^* (%)** | **GRADE** | **AMSTAR** |
| --- | --- | --- | --- | --- | --- | --- | --- | --- | --- | --- | --- |
| **Digestive system cancers** | | | | | | | | | | | |
| Deng, 2024 ^4^ | Gastric cancer | OS | Low vs. high | Overall | 30 | HR | 1.82 (1.62, 2.03) | <0.001 | 55.7 | Moderate | High |
| Deng, 2024 ^4^ | Gastric cancer | RFS | Low vs. high | Overall | 5 | HR | 2.52 (1.41, 4.47) | 0.002 | 91.2 | Moderate | High |
| Deng, 2024 ^4^ | Gastric cancer | CSS | Low vs. high | Overall | 9 | HR | 1.44 (1.24, 1.67) | <0.001 | 0 | High | High |
| Deng, 2024 ^4^ | Gastric cancer | Postoperative complications | Low vs. high | Overall | 15 | OR | 1.65 (1.30, 2.09) | <0.001 | 66.7 | Moderate | High |
| Fan, 2019 ^5^ | Hepatocellular carcinoma | OS | Low vs. high | Overall | 8 | HR | 2.27 (1.77, 2.91) | <0.001 | 36.1 | High | Moderate |
| Fan, 2019 ^5^ | Hepatocellular carcinoma | RFS | Low vs. high | Overall | 6 | HR | 1.68 (1.45, 1.94) | <0.001 | 0 | High | Moderate |
| Hou, 2024 ^6^ | Gastric or gastro-esophageal junction cancer | OS | High vs. low | Overall | 7 | HR | 0.55 (0.42, 0.73) | <0.001 | 31.7 | High | High |
| Hou, 2024 ^6^ | Gastric or gastro-esophageal junction cancer | PFS | High vs. low | Overall | 6 | HR | 0.71 (0.53, 0.94) | 0.018 | 72.6 | Low | High |
| Hou, 2024 ^6^ | Gastric or gastro-esophageal junction cancer | Objective remission rate | High vs. low | Overall | 3 | RR | 1.49 (0.95, 2.31) | 0.08 | 52.4 | Moderate | High |
| Hou, 2024 ^6^ | Gastric or gastro-esophageal junction cancer | Disease control rate | High vs. low | Overall | 3 | RR | 1.23 (1.09, 1.40) | 0.001 | 0 | High | High |
| Jiang, 2021 ^9^ | Oesophageal cancer | OS | Low vs. high | Overall | 11 | HR | 1.51 (1.36, 1.68) | <0.001 | 45.7 | Moderate | High |
| Kang, 2022 ^11^ | Gastrointestinal stromal tumours | RFS | Low vs. high | Overall | 8 | HR | 2.02 (1.66, 2.47) | <0.001 | 0 | High | Moderate |
| Li, 2018 (a) ^13^ | Gastric cancer | OS | Low vs. high | Overall | 15 | HR | 1.81 (1.56, 2.09) | <0.001 | 49.3 | High | Moderate |
| Li, 2018 (a) ^13^ | Gastric cancer | RFS | Low vs. high | Overall | 5 | HR | 1.82 (1.20, 2.77) | 0.006 | 80.5 | Very low | Moderate |
| Li, 2018 (a) ^13^ | Gastric cancer | CSS | Low vs. high | Overall | 5 | HR | 1.61 (1.24, 2.1) | <0.001 | 0 | High | Moderate |
| Li, 2018 (a) ^13^ | Gastric cancer | Postoperative complications | Low vs. high | Overall | 8 | RR | 1.77 (1.44, 2.17) | <0.001 | 61.7 | Moderate | Moderate |
| Li, 2019 (a) ^15^ | Pancreatic cancer | OS | Low vs. high | Overall | 10 | HR | 1.48 (1.32, 1.66) | <0.001 | 25.2 | High | Moderate |
| Li, 2019 (b) ^16^ | Esophageal squamous cell carcinoma | OS | Low vs. high | Overall | 8 | HR | 1.42 (1.20, 1.68) | <0.001 | 32.7 | High | High |
| Li, 2022 (a) ^17^ | Gastrointestinal stromal tumors | RFS | High vs. low | Overall | 8 | HR | 0.52 (0.40, 0.68) | <0.001 | 37.5 | High | High |
| Liao, 2019 ^20^ | Esophageal Cancer | OS | Low vs. high | Overall | 11 | HR | 1.29 (1.11, 1.50) | 0.001 | 54.1 | Low | High |
| Liao, 2019 ^20^ | Esophageal Cancer | CSS | Low vs. high | Overall | 3 | HR | 2.18 (1.68, 2.83) | <0.001 | 38.8 | High | High |
| Liu, 2025 ^22^ | Pancreatic cancer/nonpancreatic cancer/mixed | OS | Low vs. high | Overall | 15 | HR | 1.62 (1.39, 1.88) | < 0.001 | 83.7 | Very low | Moderate |
| Liu, 2025 ^22^ | Pancreatic cancer/nonpancreatic cancer/mixed | DFS | Low vs. high | Overall | 4 | HR | 1.44 (1.00, 2.07) | 0.05 | 72.8 | Low | Moderate |
| Lv, 2020 ^25^ | Biliary tract cancer | OS | Low vs. high | Overall | 7 | HR | 1.65 (1.42, 1.93) | <0.001 | 0 | High | High |
| Lv, 2020 ^25^ | Biliary tract cancer | OS | Low vs. high | Subgroup: surgery | 4 | HR | 1.59 (1.32, 1.92) | <0.001 | 0 | High | High |
| Man, 2018 ^26^ | Hepatocellular carcinoma | OS | Low vs. high | Subgroup: Surgical resection | 7 | HR | 2.16 (1.39, 3.34) | 0.001 | 89.7 | Low | Moderate |
| Man, 2018 ^26^ | Hepatocellular carcinoma /mixed | OS | Low vs. high | Subgroup: Transcatheter arterial chemoembolization or mixed | 4 | HR | 1.49 (1.35, 1.65) | <0.001 | 0 | High | Moderate |
| Man, 2018 ^26^ | Hepatocellular carcinoma | OS | Low vs. high | Overall | 11 | HR | 1.82 (1.44, 2.31) | <0.001 | 90.4 | Very low | Moderate |
| Man, 2018 ^26^ | Hepatocellular carcinoma | DFS | Low vs. high | Overall | 5 | HR | 1.49 (1.06, 2.07) | 0.02 | 90.4 | Very low | Moderate |
| Man, 2018 ^26^ | Hepatocellular carcinoma | RFS | Low vs. high | Overall | 4 | HR | 1.92 (1.33, 2.76) | <0.001 | 57.1 | Moderate | Moderate |
| Pierce, 2024 ^32^ | Colorectal cancer/Gastric Cancer | OS | Low vs. high | Overall | 4 | HR | 2.62 (1.23, 5.56) | 0.012 | 67.4 | Moderate | High |
| Ren, 2022 ^35^ | Gastrointestinal stromal tumors | RFS | Low vs. high | Subgroup: Univariate analysis | 6 | HRs | 2.73 (2.17, 3.43) | <0.001 | 0 | High | High |
| Ren, 2022 ^35^ | Gastrointestinal stromal tumors | RFS | Low vs. high | Subgroup: Multivariate analysis | 7 | HRs | 1.82 (1.46, 2.27) | <0.001 | 38.2 | High | High |
| Sun, 2019 ^39^ | Colorectal cancer | OS | Low vs. high | Overall | 10 | HR | 1.87 (1.45, 2.42) | <0.001 | 87.7 | Very low | High |
| Sun, 2019 ^39^ | Colorectal cancer | OS | Low vs. high | Subgroup: TNM I | 3 | HR | 1.88 (0.95, 3.71) | 0.069 | 50.2 | Moderate | High |
| Sun, 2019 ^39^ | Colorectal cancer | OS | Low vs. high | Subgroup: TNM II | 4 | HR | 1.93 (1.29, 2.90) | 0.001 | 71.4 | Low | High |
| Sun, 2019 ^39^ | Colorectal cancer | OS | Low vs. high | Subgroup: TNM III | 5 | HR | 1.71 (1.25, 2.34) | 0.001 | 60.9 | Low | High |
| Sun, 2019 ^39^ | Colorectal cancer | OS | Low vs. high | Subgroup: TNM IV | 3 | HR | 1.19 (0.66, 2.12) | 0.567 | 70.7 | Moderate | High |
| Sun, 2019 ^39^ | Colorectal cancer | Serious postoperative complications | Low vs. high | Overall | 3 | OR | 2.27 (1.53, 3.38) | <0.001 | 0 | High | High |
| Xue, 2019 ^51^ | Esophageal Cancer | OS | Low vs. high | Overall | 11 | HR | 1.29 (1.10, 1.50) | 0.002 | 69.8 | Low | High |
| Xue, 2019 ^51^ | Esophageal Cancer | OS | Low vs. high | Subgroup: Multivariate analysis | 10 | HR | 1.26 (1.08, 1.48) | 0.004 | 70.6 | Low | High |
| Yang, 2016 (a) ^53^ | Gastric cancer | OS | Low vs. high | Overall | 10 | HR | 1.89 (1.67, 2.13) | <0.001 | 6.9 | High | Moderate |
| Yang, 2016 (b) ^54^ | Colorectal cancer | OS | Low vs. high | Overall | 10 | HR | 1.97 (1.54, 2.53) | <0.001 | 72.1 | Low | High |
| Yang, 2016 (b) ^54^ | Colorectal cancer | CSS | Low vs. high | Overall | 4 | HR | 1.48 (1.19, 1.85) | 0.001 | 0 | High | High |
| Yu, 2024 ^55^ | Pancreatic cancer | OS | Low vs. high | Overall | 15 | HR | 1.09 (0.89, 1.32) | 0.409 | 76.3 | Low | High |
| Zeng, 2025 ^56^ | Biliary tract cancer | OS | Low vs. high | Overall | 17 | HR | 1.91 (1.59, 2.29) | <0.001 | 55.1 | Moderate | Moderate |
| Zeng, 2025 ^56^ | Biliary tract cancer | OS | Low vs. high | Subgroup: Patients with biliary tract cancer | 5 | HR | 2.72 (1.97, 3.77) | <0.001 | 0 | High | Moderate |
| Zeng, 2025 ^56^ | Biliary tract cancer | OS | Low vs. high | Subgroup: Patients with cholangiocarcinoma | 9 | HR | 1.54 (1.32, 1.81) | <0.001 | 10.9 | High | Moderate |
| Zeng, 2025 ^56^ | Biliary tract cancer | OS | Low vs. high | Subgroup: Patients with gallbladder cancer | 3 | HR | 2.25 (1.37, 3.71) | 0.001 | 81.2 | Moderate | Moderate |
| Zeng, 2025 ^56^ | Biliary tract cancer | OS | Low vs. high | Subgroup: Patients with resectable gallbladder cancer | 14 | HR | 1.84 (1.51, 2.24) | <0.001 | 54.5 | Moderate | Moderate |
| Zeng, 2025 ^56^ | Biliary tract cancer | DFS | Low vs. high | Subgroup: Advanced gallbladder cancer patients | 4 | HR | 1.93 (1.39, 2.67) | <0.001 | 0 | High | Moderate |
| Zhang, 2023 ^58^ | Gastric cancer | OS | High vs. low | Overall | 6 | HR | 0.48 (0.35, 0.65) | <0.001 | 20 | High | High |
| Zhang, 2023 ^58^ | Hepatocellular carcinoma | OS | High vs. low | Overall | 3 | HR | 0.62 (0.51, 0.76) | <0.001 | 0 | High | High |
| Zhang, 2023 ^58^ | Esophageal cancer | OS | High vs. low | Overall | 4 | HR | 0.46 (0.30, 0.71) | 0.002 | 36.6 | Moderate | High |
| Zhang, 2023 ^58^ | Gastrointestinal cancer | OS | High vs. low | Overall | 14 | HR | 0.53 (0.46, 0.62) | <0.001 | 34.4 | Moderate | High |
| Zhang, 2023 ^58^ | Gastrointestinal cancer | OS | High vs. low | Subgroup: PNI cut-off value > 45 | 3 | HR | 0.50 (0.32, 0.80) | 0.004 | 58.8 | Moderate | High |
| Zhang, 2023 ^58^ | Gastrointestinal cancer | OS | High vs. low | Subgroup: PNI 40-45 | 7 | HR | 0.47 (0.35, 0.63) | <0.001 | 39.5 | Moderate | High |
| Zhang, 2023 ^58^ | Gastrointestinal cancer | OS | High vs. low | Subgroup: PNI < 40 | 3 | HR | 0.31 (0.10, 0.96) | 0.044 | 54.6 | Low | High |
| Zhang, 2023 ^58^ | Gastrointestinal cancer | OS | High vs. low | Subgroup: Univariate analysis | 5 | HR | 0.51 (0.36, 0.72) | <0.001 | 25.8 | High | High |
| Zhang, 2023 ^58^ | Gastrointestinal cancer | OS | High vs. low | Subgroup: Multivariate analysis | 9 | HR | 0.46 (0.35, 0.61) | <0.001 | 44.1 | Moderate | High |
| Zhang, 2023 ^58^ | Gastric cancer | PFS | High vs. low | Overall | 4 | HR | 0.61 (0.45, 0.85) | 0.003 | 0 | Moderate | High |
| Zhang, 2023 ^58^ | Esophageal cancer | PFS | High vs. low | Overall | 4 | HR | 0.53 (0.33, 0.83) | 0.034 | 39.9 | Moderate | High |
| Zhang, 2023 ^58^ | Gastrointestinal cancer | PFS | High vs. low | Overall | 12 | HR | 0.74 (0.65, 0.84) | <0.001 | 22 | Moderate | High |
| Zhang, 2023 ^58^ | Gastrointestinal cancer | PFS | High vs. low | Subgroup: PNI 40-45 | 7 | HR | 0.67 (0.54, 0.84) | 0.001 | 36.5 | Moderate | High |
| Zhang, 2023 ^58^ | Gastrointestinal cancer | PFS | High vs. low | Subgroup: Multivariate analysis | 8 | HR | 0.63 (0.48, 0.81) | <0.001 | 45.5 | Moderate | High |
| Zhang, 2023 ^58^ | Gastrointestinal cancer | PFS | High vs. low | Subgroup: Univariate analysis | 4 | HR | 0.77 (0.55, 1.09) | 0.14 | 0 | Moderate | High |
| Zhang, 2024 (a) ^59^ | Hepatocellular carcinoma | OS | Low vs. high | Overall | 13 | HR | 1.70 (1.47, 1.96) | <0.001 | 37.2 | Moderate | High |
| Zhang, 2024 (a) ^59^ | Hepatocellular carcinoma | RFS | Low vs. high | Overall | 15 | HR | 1.48 (1.30, 1.69) | <0.001 | 56.3 | Moderate | High |
| Zhao, 2016 ^61^ | Gastric cancer, colorectal cancer, hepatocellular carcinoma, pancreatic cancer, esophageal carcinoma | OS | Low vs. high | Overall | 22 | HR | 1.83 (1.62,2.07) | <0.001 | 50.2 | Low | Moderate |
| Zhao, 2016 ^61^ | Gastric cancer, colorectal cancer, hepatocellular carcinoma, pancreatic cancer, esophageal carcinoma | OS | Low vs. high | Subgroup: PNI >=45 | 18 | HR | 1.74 (1.54, 1.97) | <0.001 | 48.4 | Moderate | Moderate |
| Zhao, 2016 ^61^ | Gastric cancer, colorectal cancer, hepatocellular carcinoma, pancreatic cancer, esophageal carcinoma | OS | Low vs. high | Subgroup: PNI < 45 | 4 | HR | 2.62 (1.91, 3.6) | <0.001 | 0 | High | Moderate |
| Zhao, 2016 ^61^ | Gastric carcinoma | OS | Low vs. high | Overall | 7 | HR | 2.07 (1.72, 2.49) | <0.001 | 23 | High | Moderate |
| Zhao, 2016 ^61^ | Colorectal carcinoma | OS | Low vs. high | Overall | 7 | HR | 1.93 (1.39, 2.68) | <0.001 | 69.2 | Low | Moderate |
| Zhao, 2016 ^61^ | Hepatocellular carcinoma | OS | Low vs. high | Overall | 4 | HR | 1.75 (1.32, 2.32) | <0.001 | 54.5 | Moderate | Moderate |
| Zhao, 2016 ^61^ | Pancreatic carcinoma | OS | Low vs. high | Overall | 3 | HR | 1.58 (1.28, 1.94) | <0.001 | 23 | Moderate | Moderate |
| Zhao, 2016 ^61^ | Gastric cancer, colorectal cancer, hepatocellular carcinoma, pancreatic cancer, esophageal carcinoma | DFS | Low vs. high | Overall | 3 | HR | 1.85 (1.19, 2.89) | 0.006 | 68 | Moderate | Moderate |
| Zhao, 2016 ^61^ | Gastric cancer, colorectal cancer, hepatocellular carcinoma, pancreatic cancer, esophageal carcinoma | Post-operative complications | Low vs. high | Overall | 3 | HR | 2.31 (1.63, 3.28) | <0.001 | 26.4 | High | Moderate |
| Zhao, 2016 ^61^ | Gastric cancer, colorectal cancer, hepatocellular carcinoma, pancreatic cancer, esophageal carcinoma | Post-operative complications/DFS/CSS | Low vs. high | Overall | 8 | HR | 1.98 (1.54, 2.54) | <0.001 | 62.3 | Low | Moderate |
| Zhao, 2022 ^63^ | Pancreatic cancer | OS | Low vs. high | Overall | 14 | HR | 1.66 (1.42, 1.94) | <0.001 | 43 | High | Moderate |
| **Respiratory system cancers** | | | | | | | | | | | |
| Jiang, 2020 ^8^ | Small cell lung cancer | OS | Low vs. high | Overall | 9 | HR | 1.43 (1.24, 1.64) | <0.001 | 66.8 | Moderate | High |
| Jiang, 2020 ^8^ | Small cell lung cancer | PFS | Low vs. high | Overall | 3 | HR | 1.44 (0.89, 2.31) | 0.134 | 63.3 | Moderate | High |
| Li, 2018 (b) ^14^ | Lung cancer | OS | Low vs. high | Overall | 10 | HR | 1.72 (1.43, 2.06) | <0.001 | 66.7 | Low | High |
| Li, 2018 (b) ^14^ | No-small cell lung cancer | OS | Low vs. high | Overall | 8 | HR | 1.93 (1.56, 2.37) | <0.001 | 52.7 | Low | High |
| Shao, 2022 ^36^ | Lung cancer | OS | Low vs. high | Overall | 7 | HR | 2.5 (1.44, 4.33) | 0.001 | 71.1 | High | High |
| Shao, 2022 ^36^ | Lung cancer | PFS | Low vs. high | Overall | 7 | HR | 1.94 (1.56, 2.42) | <0.001 | 5 | High | High |
| Wang, 2018 ^46^ | No-small cell lung cancer | OS | Low vs. high | Overall | 14 | HR | 1.59 (1.28, 1.96) | <0.001 | 81 | Very low | High |
| Wang, 2018 ^46^ | Small cell lung cancer | OS | Low vs. high | Overall | 4 | HR | 1.57 (1.17, 2.12) | 0.003 | 85.9 | Low | High |
| Wang, 2018 ^46^ | No-small cell lung cancer | DFS/RFS | Low vs. high | Overall | 7 | HR | 1.74 (1.08, 2.8) | 0.023 | 90.4 | Low | High |
| Wang, 2018 ^46^ | No-small cell lung cancer | RFS | Low vs. high | Overall | 3 | HR | 1.52 (1.26, 1.83) | <0.001 | 0 | High | High |
| Wang, 2024 ^48^ | Lung cancer | OS | High vs. low | Overall | 19 | HR | 0.43 (0.34, 0.54) | <0.001 | 60.6 | High | High |
| Wang, 2024 ^48^ | Lung cancer | PFS | High vs. low | Overall | 20 | HR | 0.51 (0.43, 0.61) | <0.001 | 55.6 | Moderate | High |
| Xia, 2023 ^49^ | Advanced non-small cell lung cancer | OS | Low vs. high | Overall | 11 | HR | 2.68 (1.76, 4.06) | <0.001 | 91 | Very low | High |
| Xia, 2023 ^49^ | Advanced non-small cell lung cancer | PFS | Low vs. high | Overall | 11 | HR | 1.84 (1.39, 2.42) | 0.001 | 82.9 | Low | High |
| Yan, 2023 ^52^ | Advanced lung cancer | OS | Low vs. high | Overall | 13 | HR | 2.56 (1.86, 3.54) | <0.001 | 53.5 | High | High |
| Yan, 2023 ^52^ | Advanced lung cancer | PFS | Low vs. high | Overall | 10 | HR | 1.91 (1.53, 2.4) | <0.001 | 30.4 | High | High |
| Zhang, 2021 ^57^ | Lung cancer | OS | Low vs. high | Overall | 15 | HR | 1.22 (0.89, 1.67) | 0.213 | 94.9 | Very low | Moderate |
| Zhang, 2021 ^57^ | Small lung cancer | OS | Low vs. high | Overall | 6 | HR | 0.89 (0.64, 1.24) | 0.486 | 90.9 | Very low | Moderate |
| Zhang, 2021 ^57^ | Non-small lung cancer | OS | Low vs. high | Overall | 9 | HR | 1.53 (0.97, 2.40) | 0.066 | 94.6 | Very low | Moderate |
| Zhang, 2021 ^57^ | Lung cancer | OS | Low vs. high | Subgroup: PNI cut-off value = 45 | 4 | HR | 1.14 (0.65, 1.97) | 0.654 | 94.8 | Very low | Moderate |
| Zhang, 2021 ^57^ | Lung cancer | PFS | Low vs. high | Overall | 9 | HR | 1.32 (1.02, 2.15) | 0.036 | 86.7 | Very low | Moderate |
| Zhang, 2021 ^57^ | Non-small lung cancer | PFS | Low vs. high | Overall | 7 | HR | 1.33 (0.98, 1.81) | 0.072 | 87.7 | Very low | Moderate |
| **Urinary system cancers** | | | | | | | | | | | |
| Jiao, 2023 ^10^ | Bladder Cancer | OS | Low vs. high | Overall | 6 | HR | 1.71 (1.37, 2.14) | <0.001 | 0 | High | Moderate |
| Jiao, 2023 ^10^ | Bladder Cancer | RFS | Low vs. high | Overall | 3 | HR | 1.22 (0.67, 2.24) | 0.519 | 84 | Low | Moderate |
| Kim, 2021 ^12^ | Renal Cell Carcinoma | RFS/DFS | Low vs. high | Overall | 5 | HR | 1.98 (1.57, 2.5) | <0.001 | 17.3 | High | High |
| Kim, 2021 ^12^ | Renal Cell Carcinoma | OS/CSS | Low vs. high | Overall | 8 | HR | 1.68 (1.44, 1.96) | <0.001 | 8.8 | Moderate | High |
| Mao, 2021 ^27^ | Non-metastatic Renal Cell Carcinoma | OS | Low vs. high | Overall | 3 | HR | 2.91 (1.60, 5.29) | <0.001 | 69.6 | High | High |
| Mao, 2021 ^27^ | Metastatic Renal Cell Carcinoma | OS | Low vs. high | Overall | 3 | HR | 2.02 (1.49, 2.73) | <0.001 | 52.4 | High | High |
| Mao, 2021 ^27^ | Renal Cell Carcinoma | OS | Low vs. high | Overall | 8 | HR | 2.10 (1.67, 2.64) | <0.001 | 56.8 | High | High |
| Mao, 2021 ^27^ | Clear cell renal cell carcinoma and non- clear cell renal cell carcinoma | OS | Low vs. high | Subgroup: asian | 7 | HR | 2.17 (1.68, 2.81) | <0.001 | 62.1 | High | High |
| Mao, 2021 ^27^ | Renal Cell Carcinoma | OS | Low vs. high | Subgroup: PNI < 45 | 4 | HR | 1.94 (1.54, 2.44) | <0.001 | 33.8 | High | High |
| Mao, 2021 ^27^ | Renal Cell Carcinoma | OS | Low vs. high | Subgroup: PNI >= 45 | 4 | HR | 2.42 (1.49, 3.93) | <0.001 | 72.9 | High | High |
| Mao, 2021 ^27^ | Renal Cell Carcinoma | OS | Low vs. high | Subgroup: treatment: partialorradicalnephrectomy | 4 | HR | 2.02 (1.39, 2.93) | <0.001 | 65.2 | High | High |
| Mao, 2021 ^27^ | Renal Cell Carcinoma | CSS | Low vs. high | Overall | 4 | HR | 2.95 (1.61, 5.39) | <0.001 | 67.6 | High | High |
| Mao, 2021 ^27^ | Renal Cell Carcinoma | CSS | Low vs. high | Subgroup: PNI > 45 | 3 | HR | 4.05 (2.61, 6.29) | <0.001 | 0 | High | High |
| Mao, 2021 ^27^ | Renal Cell Carcinoma | CSS | Low vs. high | Subgroup: determinationmethods | 4 | HR | 2.58 (1.87, 3.57) | <0.001 | 67.6 | High | High |
| Mao, 2021 ^27^ | Renal Cell Carcinoma | PFS/DFS/RFS | Low vs. high | Subgroup: Metastaticstatus: Non-metastatic | 3 | HR | 2.44 (1.75, 3.4) | <0.001 | 0 | High | High |
| Mao, 2021 ^27^ | Renal Cell Carcinoma | PFS/DFS/RFS | Low vs. high | Overall | 7 | HR | 1.99 (1.67, 2.36) | <0.001 | 0 | Moderate | High |
| Mao, 2021 ^27^ | Clear cell renal cell carcinoma and non- clear cell renal cell carcinoma | PFS/DFS/RFS | Low vs. high | Subgroup: Histology: clear cell renal cell carcinoma + non-clear cell renal cell carcinoma | 5 | HR | 1.94 (1.60, 2.36) | <0.001 | 10.8 | Moderate | High |
| Mao, 2021 ^27^ | Renal Cell Carcinoma | PFS/DFS/RFS | Low vs. high | Subgroup: PNI cut-off <45 | 3 | HR | 2.15 (1.60, 2.88) | <0.001 | 0 | High | High |
| Mao, 2021 ^27^ | Renal Cell Carcinoma | PFS/DFS/RFS | Low vs. high | Subgroup: PNI >=45 | 4 | HR | 1.91 (1.55, 2.36) | <0.001 | 14 | High | High |
| Mao, 2021 ^27^ | Renal Cell Carcinoma | PFS/DFS/RFS | Low vs. high | Subgroup: Treatment: Partialorradical nephrectomy | 5 | HR | 1.98 (1.62, 2.40) | <0.001 | 0 | High | High |
| Meng, 2022 ^29^ | Upper tract urothelial carcinoma | OS | Low vs. high | Overall | 5 | HR | 1.92 (1.60, 2.30) | <0.001 | 0 | Moderate | High |
| Meng, 2022 ^29^ | Upper tract urothelial carcinoma | CSS/DSS | Low vs. high | Overall | 6 | HR | 1.79 (1.49, 2.16) | <0.001 | 0 | High | High |
| Meng, 2022 ^29^ | Upper tract urothelial carcinoma | PFS/DFS/RFS | Low vs. high | Overall | 4 | HR | 1.57 (1.33, 1.85) | <0.001 | 0 | High | High |
| Qi, 2018 ^34^ | Urinary cancers | OS | Low vs. high | Overall | 9 | HR | 1.68 (1.45, 1.95) | <0.001 | 0 | High | High |
| Qi, 2018 ^34^ | Urinary cancers | OS | Low vs. high | Subgroup: Treatment type: target therapy | 3 | HR | 1.88 (1.34, 2.63) | <0.001 | 0 | High | High |
| Qi, 2018 ^34^ | Urinary cancers | OS | Low vs. high | Subgroup: Treatment type: Surgery | 6 | HR | 1.64 (1.4, 1.93) | <0.001 | 0 | High | High |
| Qi, 2018 ^34^ | Renal cell cancer | OS | Low vs. high | Overall | 5 | HR | 1.65 (1.37, 1.97) | <0.001 | 0 | Moderate | High |
| Qi, 2018 ^34^ | Urinary cancers | CSS/DSS | Low vs. high | Overall | 5 | HR | 1.57 (1.33, 1.86) | <0.001 | 40.7 | High | High |
| Qi, 2018 ^34^ | Urinary cancers | PFS/DFS/RFS | Low vs. high | Overall | 10 | HR | 1.75 (1.53, 1.99) | <0.001 | 0 | High | High |
| Qi, 2018 ^34^ | Urinary cancer | PFS/DFS/RFS | Low vs. high | Subgroup: Treatment type: target therapy | 3 | HR | 2.14 (1.5, 3.05) | <0.001 | 6.6 | High | High |
| Qi, 2018 ^34^ | Urinary cancer | PFS/DFS/RFS | Low vs. high | Subgroup: Treatment type: Surgery | 7 | HR | 1.69 (1.47, 1.95) | <0.001 | 0 | High | High |
| Qi, 2018 ^34^ | Renal cell cancer | PFS/DFS/RFS | Low vs. high | Overall | 6 | HR | 1.81 (1.54, 2.13) | <0.001 | 0 | Moderate | High |
| Sun, 2025 ^40^ | Bladder cancer | OS | Low vs. high | Overall | 10 | HR | 1.8 (1.54, 2.10) | <0.001 | 0 | High | High |
| Sun, 2025 ^40^ | Bladder cancer | RFS | Low vs. high | Overall | 7 | HR | 1.53 (1.15, 2.04) | 0.003 | 70.7 | Moderate | High |
| Tang, 2021 ^43^ | Renal Cell Carcinoma | OS | normal vs. low | Overall | 6 | HR | 1.57 (1.37, 1.8) | <0.001 | 0 | High | Moderate |
| Tang, 2021 ^43^ | Renal Cell Carcinoma | RFS | normal vs. low | Overall | 6 | HR | 1.69 (1.45, 1.96) | <0.001 | 0 | Moderate | Moderate |
| Zhao, 2019 ^62^ | Urologic tumors | OS | Low vs. high | Overall | 9 | HR | 1.72 (1.48, 2.00) | <0.001 | 0 | Moderate | Moderate |
| Zhao, 2019 ^62^ | Urologic tumors | OS | Low vs. high | Subgroup: PNI > 50 | 3 | HR | 1.60 (1.20, 2.13) | 0.001 | 0 | High | Moderate |
| Zhao, 2019 ^62^ | Urologic tumors | OS | Low vs. high | Subgroup: PNI <50 | 6 | HR | 1.77 (1.48, 2.11) | <0.001 | 1.1 | Moderate | Moderate |
| **Reproductive system cancers** | | | | | | | | | | | |
| Cao, 2024 ^2^ | Cervical cancer | OS | Low vs. high | Subgroup: Univariate analysis | 9 | HR | 1.38 (0.77, 2.49) | 0.277 | 88.3 | Low | High |
| Cao, 2024 ^2^ | Cervical cancer | OS | Low vs. high | Subgroup: Multivariate analysis | 7 | HR | 1.06 (0.64, 1.76) | 0.828 | 82.6 | Low | High |
| Cao, 2024 ^2^ | Cervical cancer | OS | Low vs. high | Subgroup: Univariate: region: China | 7 | HR | 0.87 (0.47, 1.61) | 0.664 | 83 | Low | High |
| Cao, 2024 ^2^ | Cervical cancer | OS | Low vs. high | Subgroup: Univariate: region: Japan | 3 | HR | 3.75 (2.45, 5.74) | 0.138 | 0 | High | High |
| Cao, 2024 ^2^ | Cervical cancer | OS | Low vs. high | Subgroup: univariate: pni < 48 | 4 | HR | 1.26 (0.49, 3.26) | 0.635 | 88.7 | Low | High |
| Cao, 2024 ^2^ | Cervical cancer | OS | Low vs. high | Subgroup: univariate: pni > 48 | 5 | HR | 1.51 (0.54, 4.27) | 0.435 | 89.8 | Low | High |
| Cao, 2024 ^2^ | Cervical cancer | OS | Low vs. high | Subgroup: Univariate: sample size < 150 | 4 | HR | 1.77 (0.73, 4.33) | 0.209 | 91 | Low | High |
| Cao, 2024 ^2^ | Cervical cancer | OS | Low vs. high | Subgroup: Univariate: sample size > 150 | 5 | HR | 1.10 (0.37, 3.22) | 0.868 | 87.4 | Low | High |
| Cao, 2024 ^2^ | Cervical cancer | OS | Low vs. high | Subgroup: Univariate: treatment: radiotherapy | 3 | HR | 1.98 (0.71, 5.55) | 0.195 | 86.8 | Very low | High |
| Cao, 2024 ^2^ | Cervical cancer | OS | Low vs. high | Subgroup: Univariate: treatment: surgery | 4 | HR | 0.87 (0.24, 3.16) | 0.837 | 83.2 | Low | High |
| Cao, 2024 ^2^ | Cervical cancer | OS | Low vs. high | Subgroup: Multivariate: Region: China | 5 | HR | 0.64 (0.36, 1.14) | 0.131 | 80.3 | Low | High |
| Cao, 2024 ^2^ | Cervical cancer | OS | Low vs. high | Subgroup: multivariate: region: Japan | 3 | HR | 2.95 (1.86, 4.67) | <0.001 | 0 | High | High |
| Cao, 2024 ^2^ | Cervical cancer | OS | Low vs. high | Subgroup: multivariate: pni < 48 | 3 | HR | 1.06 (0.37, 2.99) | 0.920 | 88.2 | Low | High |
| Cao, 2024 ^2^ | Cervical cancer | OS | Low vs. high | Subgroup: multivariate: pni > 48 | 5 | HR | 1.03 (0.45, 2.35) | 0.954 | 82.5 | Low | High |
| Cao, 2024 ^2^ | Cervical cancer | OS | Low vs. high | Subgroup: Multivariate: sample size < 150 | 5 | HR | 1.48 (0.70, 3.13) | 0.305 | 84.2 | Low | High |
| Cao, 2024 ^2^ | Cervical cancer | OS | Low vs. high | Subgroup: Multivariate: sample size > 150 | 3 | HR | 0.62 (0.27, 1.43) | 0.260 | 77.3 | Low | High |
| Cao, 2024 ^2^ | Cervical cancer | OS | Low vs. high | Subgroup: Multivariate: treatment: radiotherapy | 3 | HR | 1.06 (1.02, 1.10) | 0.002 | 0 | Moderate | High |
| Cao, 2024 ^2^ | Cervical cancer | OS | Low vs. high | Subgroup: univariate: nos = 6 | 4 | HR | 2.46 (1.06, 5.70) | 0.035 | 87.7 | High | High |
| Cao, 2024 ^2^ | Cervical cancer | OS | Low vs. high | Subgroup: univariate: nos = 7 | 3 | HR | 0.33 (0.21, 0.51) | <0.001 | 0 | Low | High |
| Cao, 2024 ^2^ | Cervical cancer | OS | Low vs. high | Subgroup: multivariate: nos = 7 | 3 | HR | 0.38 (0.24, 0.61) | <0.001 | 0 | High | High |
| Cao, 2024 ^2^ | Cervical cancer | PFS | Low vs. high | Subgroup: Univariate analysis | 5 | HR | 1.11 (0.43, 2.86) | 0.823 | 90 | Low | High |
| Cao, 2024 ^2^ | Cervical cancer | PFS | Low vs. high | Subgroup: Multivariate analysis | 3 | HR | 1.22 (0.65, 2.30) | 0.535 | 81.8 | Low | High |
| Cao, 2024 ^2^ | Cervical cancer | PFS | Low vs. high | Univariate: region: China | 4 | HR | 0.60 (0.26, 1.38) | 0.232 | 82.3 | Very low | High |
| Cao, 2024 ^2^ | Cervical cancer | PFS | Low vs. high | Subgroup: univariate: pni > 48 | 4 | HR | 1.45 (0.51, 4.12) | 0.487 | 89.4 | Low | High |
| Cao, 2024 ^2^ | Cervical cancer | PFS | Low vs. high | Subgroup: Univariate: sample size < 150 | 3 | HR | 1.64 (0.29, 9.17) | 0.576 | 93.7 | Low | High |
| Cao, 2024 ^2^ | Cervical cancer | PFS | Low vs. high | Subgroup: Univariate: sample size > 150 | 3 | HR | 0.77 (0.25, 2.35) | 0.643 | 85.6 | Very low | High |
| Cao, 2024 ^2^ | Cervical cancer | PFS | Low vs. high | Subgroup: univariate: nos = 6 | 3 | HR | 1.87 (0.49, 7.11) | 0.360 | 91.4 | Low | High |
| Li, 2024 ^19^ | Ovarian cancer/Cervical cancer patients/endometrial cancer | OS | Low vs. high | Overall | 26 | HR | 1.60 (1.39, 1.84) | <0.001 | 80 | Very low | High |
| Li, 2024 ^19^ | Ovarian cancer/Cervical cancer patients/Endometrial cancer | PFS | Low vs. high | Overall | 11 | HR | 1.63 (1.20, 2.23) | 0.002 | 78.8 | Low | High |
| Li, 2024 ^19^ | Ovarian cancer/Cervical cancer patients/Endometrial cancer | DFS | Low vs. high | Overall | 6 | HR | 1.73 (1.19, 2.52) | 0.004 | 91.3 | Very low | High |
| Mao, 2024 ^28^ | Endometrial cancer | OS | Low vs. high | Overall | 8 | HR | 1.72 (1.33, 2.21) | <0.001 | 79.8 | Very low | High |
| Mao, 2024 ^28^ | Endometrial cancer | PFS | Low vs. high | Overall | 5 | HR | 2.49 (1.62, 3.85) | <0.001 | 76.1 | Moderate | High |
| Niu, 2023 ^31^ | Cervical cancer | OS | Low vs. high | Overall | 8 | HR | 2.98 (2.22, 3.99) | <0.001 | 5.6 | High | High |
| Niu, 2023 ^31^ | Cervical cancer | PFS | Low vs. high | Overall | 6 | HR | 2.43 (1.92, 3.07) | <0.001 | 35.3 | High | High |
| Tan, 2022 ^41^ | Ovarian cancer | OS | High vs. low | Overall | 10 | HR | 0.67 (0.53, 0.84) | <0.001 | 87.3 | Low | High |
| Tan, 2022 ^41^ | Ovarian cancer | PFS | High vs. low | Overall | 6 | HR | 0.74 (0.63, 0.87) | <0.001 | 86 | Very low | High |
| Tobing, 2024 ^44^ | Prostate cancer | OS | Low vs. high | Overall | 9 | HR | 1.99 (1.45, 2.72) | <0.001 | 71.1 | Low | High |
| Tobing, 2024 ^44^ | Prostate cancer | PFS | Low vs. high | Overall | 10 | HR | 1.97 (1.55, 2.51) | <0.001 | 48.6 | Moderate | High |
| Wang, 2019 ^47^ | Gynecological cancers | OS | Low vs. high | Subgroup: Univariate analysis | 8 | HR | 2.66 (1.56, 4.55) | <0.001 | 87 | Low | High |
| Wang, 2019 ^47^ | Gynecological cancers | OS | Low vs. high | Subgroup: Multivariate analysis | 4 | HR | 1.88 (1.10, 3.20) | 0.021 | 89.1 | Very low | High |
| Wang, 2019 ^47^ | Gynecological cancers | PFS | Low vs. high | Subgroup: Univariate analysis | 5 | HR | 2.43 (2.07, 2.86) | <0.001 | 0 | High | High |
| Wang, 2019 ^47^ | Gynecological cancers | PFS | Low vs. high | Subgroup: Multivariate analysis | 3 | HR | 1.92 (1.52, 2.44) | <0.001 | 0 | High | High |
| Zhang, 2024 (b) ^60^ | Endometrial cancer | OS | Low vs. high | Overall | 10 | HR | 2.01 (1.62, 2.49) | <0.001 | 54.3 | Moderate | High |
| Zhang, 2024 (b) ^60^ | Endometrial cancer | OS | Low vs. high | Subgroup: Study country: Asian | 8 | HR | 1.97 (1.55, 2.51) | <0.001 | 58.5 | Low | High |
| Zhang, 2024 (b) ^60^ | Endometrial cancer | OS | Low vs. high | Subgroup: Mean age of the patients < 56 | 5 | HR | 2.07 (1.43, 3.00) | <0.001 | 71.3 | Moderate | High |
| Zhang, 2024 (b) ^60^ | Endometrial cancer | OS | Low vs. high | Subgroup: Mean age of the patients >= 56 | 5 | HR | 1.96 (1.49, 2.57) | <0.001 | 58.5 | Moderate | High |
| Zhang, 2024 (b) ^60^ | Endometrial cancer | OS | Low vs. high | Subgroup: Patients with stage IV Endometrial cancer included | 7 | HR | 1.71 (1.40, 2.10) | <0.001 | 38.5 | Moderate | High |
| Zhang, 2024 (b) ^60^ | Endometrial cancer | OS | Low vs. high | Subgroup: Patients with stage IV EC not included | 3 | HR | 2.78 (2.01, 3.86) | <0.001 | 0 | High | High |
| Zhang, 2024 (b) ^60^ | Endometrial cancer | OS | Low vs. high | Subgroup: PNI <51 | 5 | HR | 1.75 (1.47, 2.07) | <0.001 | 0 | High | High |
| Zhang, 2024 (b) ^60^ | Endometrial cancer | OS | Low vs. high | Subgroup: PNI >=51 | 5 | HR | 2.40 (1.51, 3.82) | <0.001 | 76.2 | Low | High |
| Zhang, 2024 (b) ^60^ | Endometrial cancer | OS | Low vs. high | Subgroup: Follow-up duration < 60 months | 5 | HR | 2.26 (1.63, 3.12) | <0.001 | 52.6 | Moderate | High |
| Zhang, 2024 (b) ^60^ | Endometrial cancer | OS | Low vs. high | Subgroup: Follow-up duration >= 60 months | 5 | HR | 1.83 (1.32, 2.52) | <0.001 | 52.1 | Moderate | High |
| Zhang, 2024 (b) ^60^ | Endometrial cancer | PFS | Low vs. high | Overall | 6 | HR | 2.75 (1.74, 4.33) | <0.001 | 78.2 | Low | High |
| Zhang, 2024 (b) ^60^ | Endometrial cancer | PFS | Low vs. high | Subgroup: Study country: Asian | 4 | HR | 2.76 (1.56, 4.89) | <0.001 | 82.2 | Moderate | High |
| Zhang, 2024 (b) ^60^ | Endometrial cancer | PFS | Low vs. high | Subgroup: Mean age of the patients < 60 | 3 | HR | 3.46 (1.98, 6.03) | <0.001 | 59.8 | High | High |
| Zhang, 2024 (b) ^60^ | Endometrial cancer | PFS | Low vs. high | Subgroup: Mean age of the patients >= 60 | 3 | HR | 2.17 (1.20, 3.94) | 0.011 | 75 | High | High |
| Zhang, 2024 (b) ^60^ | Endometrial cancer | PFS | Low vs. high | Subgroup: Patients with stage IV Endometrial cancer included | 4 | HR | 2.88 (1.43, 5.78) | 0.003 | 85.4 | Moderate | High |
| Zhang, 2024 (b) ^60^ | Endometrial cancer | PFS | Low vs. high | Subgroup: Follow-up duration < 48 months | 3 | HR | 3.11 (1.47, 6.60) | 0.003 | 76.2 | Moderate | High |
| Zhang, 2024 (b) ^60^ | Endometrial cancer | PFS | Low vs. high | Subgroup: Follow-up duration >= 48 months | 3 | HR | 2.45 (1.35, 4.45) | 0.003 | 78.1 | Moderate | High |
| Zheng, 2023 ^64^ | Prostate cancer | OS | Low vs. high | Overall | 7 | HR | 2.16 (1.40, 3.34) | 0.001 | 74.9 | Moderate | High |
| Zheng, 2023 ^64^ | Prostate cancer | OS | Low vs. high | Subgroup: Disease: mcrpc | 3 | HR | 2.90 (1.81, 4.63) | <0.001 | 0 | High | High |
| Zheng, 2023 ^64^ | Prostate cancer | OS | Low vs. high | Subgroup: Disease: nmcrpc | 4 | HR | 1.70 (1.08, 2.68) | 0.021 | 69.8 | Moderate | High |
| Zheng, 2023 ^64^ | Prostate cancer | PFS | Low vs. high | Overall | 8 | HR | 2.17 (1.63, 2.89) | <0.001 | 51.5 | High | High |
| Zheng, 2023 ^64^ | Prostate cancer | PFS | Low vs. high | Subgroup: Disease: mcrpc | 3 | HR | 3.43 (2.06, 5.71) | <0.001 | 35.3 | High | High |
| Zheng, 2023 ^64^ | Prostate cancer | PFS | Low vs. high | Subgroup: Disease: nmcrpc | 5 | HR | 1.77 (1.40, 2.23) | <0.001 | 10.1 | High | High |
| **Head and neck cancers** | | | | | | | | | | | |
| Dai, 2023 ^3^ | Oral cancer | OS | Low vs. high | Overall | 9 | HR | 2.44 (1.45, 4.12) | 0.001 | 86.7 | Low | Moderate |
| Dai, 2023 ^3^ | Oral Cancer | DFS | Low vs. high | Overall | 6 | HR | 1.92 (1.53, 2.42) | <0.001 | 24.4 | Moderate | Moderate |
| Luan, 2021 ^24^ | Head and neck cancer | OS | Low vs. high | Overall | 13 | HR | 1.93 (1.62, 2.30) | <0.001 | 41.7 | Moderate | High |
| Luan, 2021 ^24^ | Nasopharynx | OS | Low vs. high | Overall | 6 | HR | 1.86 (1.54, 2.23) | <0.001 | 0 | Low | High |
| Luan, 2021 ^24^ | Oral Cancer | OS | Low vs. high | Overall | 2 | HR | 1.77 (1.13, 2.76) | 0.013 | 71.9 | High | High |
| Luan, 2021 ^24^ | Head and neck cancer | PFS | Low vs. high | Overall | 4 | HR | 1.51 (1.19, 1.92) | 0.001 | 25.3 | High | High |
| Luan, 2021 ^24^ | Head and neck cancer | DMFS | Low vs. high | Overall | 6 | HR | 2.04 (1.74, 2.38) | <0.001 | 0 | High | High |
| Luan, 2021 ^24^ | Head and neck cancer | DSS | Low vs. high | Overall | 3 | HR | 2.20 (1.66, 2.91) | <0.001 | 0 | Low | High |
| Luan, 2021 ^24^ | Head and neck cancer | DFS | Low vs. high | Overall | 3 | HR | 1.98 (1.12, 3.50) | 0.019 | 83.8 | High | High |
| Shi, 2021 ^37^ | Head and neck neoplasms | OS | Low vs. high | Overall | 9 | HR | 1.97 (1.64, 2.37) | <0.001 | 22.8 | Moderate | High |
| Shi, 2021 ^37^ | Head and neck neoplasms | PFS | Low vs. high | Overall | 4 | HR | 1.50 (1.22, 1.84) | <0.001 | 0 | High | High |
| Shi, 2021 ^37^ | Head and neck neoplasms | Distant metastasis-free survival | Low vs. high | Overall | 5 | HR | 1.96 (1.60, 2.40) | <0.001 | 0 | High | High |
| Tang, 2020 ^42^ | Nasopharyngeal carcinoma | OS | Low vs. high | Subgroup: Univariate analysis | 5 | HR | 2.06 (1.61, 2.64) | <0.001 | 0 | High | Moderate |
| Tang, 2020 ^42^ | Nasopharyngeal carcinoma | OS | Low vs. high | Subgroup: Multivariate analysis | 6 | HR | 1.78 (1.46, 2.18) | <0.001 | 0 | High | Moderate |
| Tang, 2020 ^42^ | Nasopharyngeal carcinoma | PFS | Low vs. high | Subgroup: Univariate analysis | 3 | HR | 2.27 (1.27, 4.05) | 0.006 | 67.8 | High | Moderate |
| Tang, 2020 ^42^ | Nasopharyngeal carcinoma | PFS | Low vs. high | Subgroup: Multivariate analysis | 4 | HR | 1.45 (1.18, 1.78) | 0.001 | 7.3 | High | Moderate |
| Tang, 2020 ^42^ | Nasopharyngeal carcinoma | DMFS | Low vs. high | Subgroup: Univariate analysis | 3 | HR | 2.06 (1.60, 2.67) | <0.001 | 0 | High | Moderate |
| Tang, 2020 ^42^ | Nasopharyngeal carcinoma | DMFS | Low vs. high | Subgroup: Multivariate analysis | 4 | HR | 2.04 (1.66, 2.50) | <0.001 | 0 | High | Moderate |
| Tu, 2020 ^45^ | Nasopharyngeal carcinoma | OS | Low vs. high | Overall | 9 | HR | 1.89 (1.59, 2.25) | <0.001 | 0 | Moderate | High |
| Tu, 2020 ^45^ | Nasopharyngeal carcinoma | PFS | Low vs. high | Overall | 5 | HR | 1.59 (1.32, 1.91) | <0.001 | 48.9 | High | High |
| Tu, 2020 ^45^ | Nasopharyngeal carcinoma | DMFS | Low vs. high | Overall | 6 | HR | 2.01 (1.66, 2.43) | <0.001 | 0 | High | High |
| Tu, 2020 ^45^ | Nasopharyngeal carcinoma | RFS | Low vs. high | Overall | 3 | HR | 1.51 (1.04, 2.21) | 0.032 | 0 | High | High |
| **Hematologic system cancers** | | | | | | | | | | | |
| Luan, 2020 ^23^ | Diffuse large b-cell lymphoma | OS | Low vs. high | Overall | 7 | HR | 2.14 (1.66, 2.75) | <0.001 | 41.1 | High | High |
| Luan, 2020 ^23^ | Diffuse large b-cell lymphoma | PFS | Low vs. high | Overall | 4 | HR | 1.75 (1.36, 2.25) | 0.001 | 38.9 | High | High |
| **Nervous system cancers** | | | | | | | | | | | |
| Hung, 2023 ^7^ | Glioma | OS | High vs. low | Overall | 13 | HR | 0.61 (0.52, 0.72) | <0.001 | 25.3 | High | High |
| Hung, 2023 ^7^ | Glioma | OS | High vs. low | Subgroup: Multivariate | 10 | HR | 0.62 (0.50, 0.76) | <0.001 | 41.8 | High | High |
| Hung, 2023 ^7^ | Glioma | OS | High vs. low | Subgroup: Univariate | 3 | HR | 0.58 (0.41, 0.82) | 0.002 | 0 | High | High |
| Hung, 2023 ^7^ | Glioma | PFS | High vs. low | Overall | 4 | HR | 0.71 (0.58, 0.88) | 0.001 | 0 | High | High |
| Liu, 2020 ^21^ | Glioma | OS | High vs. low | Subgroup: Cancer type: glioblastoma multiforme | 5 | HR | 0.87 (0.61, 1.23) | 0.420 | 66.2 | Moderate | Moderate |
| Liu, 2020 ^21^ | Glioma | OS | High vs. low | Overall | 7 | HR | 0.83 (0.59, 1.16) | 0.282 | 84.5 | Low | Moderate |
| Liu, 2020 ^21^ | Glioma | OS | High vs. low | Subgroup: Univariate | 3 | HR | 1.31 (0.75, 2.27) | 0.346 | 89.9 | Low | Moderate |
| Liu, 2020 ^21^ | Glioma | OS | High vs. low | Subgroup: Multivariate | 5 | HR | 0.63 (0.52, 0.77) | <0.001 | 0 | High | Moderate |
| **Breast cancers** | | | | | | | | | | | |
| Prasetiyo, 2023 ^33^ | Breast cancer | OS | High vs. low | Overall | 9 | HR | 0.38 (0.28, 0.51) | <0.001 | 28.1 | High | High |
| Prasetiyo, 2023 ^33^ | Breast cancer | DFS | High vs. low | Overall | 7 | HR | 0.60 (0.33, 1.10) | 0.099 | 78.6 | Low | High |
| **Other cancers** | | | | | | | | | | | |
| Bullock, 2020 ^1^ | Cancer | OS | Low vs. high | Overall | 4 | HR | 1.89 (1.03, 3.48) | 0.041 | 65.2 | Moderate | Moderate |
| Li, 2022 (b) ^18^ | Advanced cancers | OS | Low vs. high | Overall | 9 | HR | 2.31 (1.81, 2.94) | <0.001 | 33 | High | High |
| Li, 2022 (b) ^18^ | Advanced cancers | PFS | Low vs. high | Overall | 8 | HR | 1.75 (1.40, 2.18) | <0.001 | 0 | High | High |
| Ni, 2022 ^30^ | Advanced-stage cancer | OS | Low vs. high | Subgroup: PNI cut-off <= 40 | 5 | HR | 2.29 (1.52, 3.44) | <0.001 | 0 | Moderate | Moderate |
| Ni, 2022 ^30^ | Advanced-stage cancer | OS | Low vs. high | Subgroup: PNI cut-off > 40 | 7 | HR | 2.14 (1.30, 3.53) | 0.003 | 76.9 | High | Moderate |
| Ni, 2022 ^30^ | Advanced-stage cancer | OS | Low vs. high | Subgroup: Multivariate | 8 | HR | 2.45 (1.59, 3.77) | <0.001 | 73.6 | High | Moderate |
| Ni, 2022 ^30^ | Advanced-stage cancer | OS | Low vs. high | Subgroup: Univariate | 4 | HR | 2.16 (1.34, 3.49) | 0.002 | 0 | High | Moderate |
| Ni, 2022 ^30^ | Advanced-stage cancer: gastrointestinal cancer | OS | Low vs. high | Overall | 3 | HR | 2.24 (1.33, 3.78) | 0.003 | 18 | High | Moderate |
| Ni, 2022 ^30^ | Advanced-stage cancer: gastrointestinal cancer | PFS | Low vs. high | Overall | 3 | HR | 1.64 (1.1, 2.45) | 0.054 | 33.8 | Moderate | Moderate |
| Ni, 2022 ^30^ | Advanced-stage cancer | PFS | Low vs. high | Subgroup: Patients treated with immune checkpoint inhibitors | 10 | HR | 1.61 (1.37, 1.88) | <0.001 | 31.4 | Moderate | Moderate |
| Ni, 2022 ^30^ | Advanced-stage cancer | PFS | Low vs. high | Subgroup: PNI cut-off <= 40 | 4 | HR | 1.64 (1.13, 2.38) | 0.010 | 0.9 | Moderate | Moderate |
| Ni, 2022 ^30^ | Advanced-stage cancer | PFS | Low vs. high | Subgroup: PNI cut-off > 40 | 6 | HR | 1.60 (1.35, 1.90) | <0.001 | 50.4 | Moderate | Moderate |
| Ni, 2022 ^30^ | Advanced-stage cancer | PFS | Low vs. high | Subgroup: Multivariate | 7 | HR | 1.62 (1.37, 1.90) | <0.001 | 51.4 | High | Moderate |
| Ni, 2022 ^30^ | Advanced-stage cancer | PFS | Low vs. high | Subgroup: Univariate | 3 | HR | 1.52 (0.87, 2.63) | 0.139 | 0 | High | Moderate |
| Ni, 2022 ^30^ | Advanced-stage cancer | Disease control rate | Low vs. high | Overall | 6 | OR | 2.48 (1.87, 3.29) | <0.001 | 24 | High | Moderate |
| Ni, 2022 ^30^ | Advanced-stage cancer | Objective response rate | Low vs. high | Overall | 12 | HR | 2.24 (1.57, 3.20) | <0.001 | 63.4 | High | Moderate |
| Ni, 2022 ^30^ | Lung cancer | OS | Low vs. high | Overall | 7 | HR | 2.36 (1.28, 4.35) | 0.006 | 72.4 | High | Moderate |
| Ni, 2022 ^30^ | Lung cancer | PFS | Low vs. high | Overall | 5 | HR | 1.95 (1.51, 2.52) | <0.001 | 24.6 | High | Moderate |
| Sun, 2014 ^38^ | Colorectal cancer | OS | Low vs. high | Overall | 12 | OR | 1.77 (1.55, 2.01) | <0.001 | 18.4 | Moderate | Moderate |
| Sun, 2014 ^38^ | Cancer | OS | Low vs. high | Overall | 13 | OR | 1.80 (1.59, 2.04) | <0.001 | 18.4 | Moderate | Moderate |
| Sun, 2014 ^38^ | Cancer | OS | Low vs. high | Subgroup: PNI >= 45 | 9 | OR | 1.67 (1.45, 1.92) | <0.001 | 0 | High | Moderate |
| Sun, 2014 ^38^ | Cancer | OS | Low vs. high | Subgroup: PNI < 45 | 4 | OR | 2.56 (1.90, 3.46) | <0.001 | 0 | High | Moderate |
| Sun, 2014 ^38^ | Gastric cancer | OS | Low vs. high | Overall | 3 | OR | 2.26 (1.63, 3.13) | <0.001 | 0 | High | Moderate |
| Xu, 2023 ^50^ | Cancer | OS | Low vs. high | Overall | 22 | HR | 2.26 (1.81, 2.82) | <0.001 | 55.7 | High | High |
| Xu, 2023 ^50^ | Cancer | PFS | Low vs. high | Overall | 19 | HR | 1.75 (1.54, 1.99) | <0.001 | 5.8 | High | High |
| Xu, 2023 ^50^ | Cancer | Disease control rate | Low vs. high | Overall | 10 | HR | 0.43 (0.34, 0.56) | <0.001 | 19.9 | High | High |

CSS, cancer-specific survival; DFS, disease-free survival; DMFS, distant metastasis-free survival; HR, hazard ratio; MVA, multivariate analyses; NA, Not Applicable; nmCRPC, nonmetastatic castration-resistant prostate cancer; NOS, Newcastle-Ottawa Scale; OR odd ratio; OS, overall survival; PFS, progression-free survival; PNI, prognostic nutritional index; RFS, recurrence-free survival; RR, relative risk; TNM Tumor-Node-Metastasis; UVA, univariate analyses.

**Supplementary table 5. The results of GRADE assessment of the evidence** **certainty of prognostic nutritional index with cancer prognostic outcomes**

| **Author, year, ref** | **Cancer site** | **Outcomes** | **Downgrade factors** | | | | | **Upgrade factors** | | | **GRADE** |
| --- | --- | --- | --- | --- | --- | --- | --- | --- | --- | --- | --- |
|  |  |  | **Risk of bias** | **Indirectness** | **Inconsistency** | **Imprecision** | **Publication bias** | **Large effect** | **Dose-response** | **Plausible confounding** |  |
| **Digestive system cancers** | | | | | | | | | | | |
| Deng, 2024 ^4^ | Gastric cancer | OS | No serious | No serious | Serious limitation ^c^ | No serious | No serious | No | No | No | Moderate |
| Deng, 2024 ^4^ | Gastric cancer | RFS | No serious | No serious | Serious limitation ^b^ | No serious | No serious | Yes ^d^ | No | No | Moderate |
| Deng, 2024 ^4^ | Gastric cancer | CSS | No serious | No serious | No serious | No serious | No serious | No | No | No | High |
| Deng, 2024 ^4^ | Gastric cancer | Postoperative complications | No serious | No serious | Serious limitation ^c^ | No serious | No serious | No | No | No | Moderate |
| Fan, 2019 ^5^ | Hepatocellular carcinoma | OS | No serious | No serious | No serious | No serious | No serious | Yes ^d^ | No | No | High |
| Fan, 2019 ^5^ | Hepatocellular carcinoma | RFS | No serious | No serious | No serious | No serious | No serious | No | No | No | High |
| Hou, 2024 ^6^ | Gastric or gastro-esophageal junction cancer | OS | No serious | No serious | No serious | No serious | No serious | No | No | No | High |
| Hou, 2024 ^6^ | Gastric or gastro-esophageal junction cancer | PFS | No serious | No serious | Serious limitation ^c^ | No serious | Serious limitation ^f^ | No | No | No | Low |
| Hou, 2024 ^6^ | Gastric or gastro-esophageal junction cancer | Objective remission rate | No serious | No serious | Serious limitation ^c^ | No serious | No serious | No | No | No | Moderate |
| Hou, 2024 ^6^ | Gastric or gastro-esophageal junction cancer | Disease control rate | No serious | No serious | No serious | No serious | No serious | No | No | No | High |
| Jiang, 2021 ^9^ | Oesophageal cancer | OS | No serious | No serious | No serious | No serious | Serious limitation ^f^ | No | No | No | Moderate |
| Kang, 2022 ^11^ | Gastrointestinal stromal tumours | RFS | No serious | No serious | No serious | No serious | No serious | Yes ^d^ | No | No | High |
| Li, 2018 (a) ^13^ | Gastric cancer after gastrectomy | OS | No serious | No serious | No serious | No serious | No serious | No | No | No | High |
| Li, 2018 (a) ^13^ | Gastric cancer | RFS | No serious | No serious | Serious limitation ^b^ | No serious | Serious limitation ^f^ | No | No | No | Very low |
| Li, 2018 (a) ^13^ | Gastric cancer | CSS | No serious | No serious | No serious | No serious | No serious | No | No | No | High |
| Li, 2018 (a) ^13^ | Gastric cancer | Postoperative complications | No serious | No serious | Serious limitation ^c^ | No serious | No serious | No | No | No | Moderate |
| Li, 2019 (a) ^15^ | Pancreatic Cancer | OS | No serious | No serious | No serious | No serious | No serious | No | No | No | High |
| Li, 2019 (b) ^16^ | Esophageal squamous cell carcinoma | OS | No serious | No serious | No serious | No serious | No serious | No | No | No | High |
| Li, 2022 (a) ^17^ | Gastrointestinal stromal tumours | RFS | No serious | No serious | No serious | No serious | No serious | No | No | No | High |
| Liao, 2019 ^20^ | Esophageal Cancer | OS | No serious | No serious | Serious limitation ^c^ | No serious | Serious limitation ^f^ | No | No | No | Low |
| Liao, 2019 ^20^ | Esophageal Cancer | CSS | No serious | No serious | No serious | No serious | Serious limitation ^f^ | Yes ^d^ | No | No | High |
| Liu, 2025 ^22^ | Pancreatic cancer /nonpancreatic cancer / mixed | OS | No serious | No serious | Serious limitation ^b^ | No serious | Serious limitation ^f^ | No | No | No | Very low |
| Liu, 2025 ^22^ | Pancreatic cancer /nonpancreatic cancer / mixed | DFS | No serious | No serious | Serious limitation ^c^ | No serious | Serious limitation ^f^ | No | No | No | Low |
| Lv, 2020 ^25^ | Biliary tract cancer | OS | No serious | No serious | No serious | No serious | No serious | No | No | No | High |
| Lv, 2020 ^25^ | Biliary tract cancer | OS | No serious | No serious | No serious | No serious | No serious | No | No | No | High |
| Man, 2018 ^26^ | Hepatocellular carcinoma | OS | No serious | No serious | Serious limitation ^b^ | No serious | Serious limitation ^f^ | Yes ^d^ | No | No | Low |
| Man, 2018 ^26^ | Hepatocellular carcinoma | OS | No serious | No serious | No serious | No serious | No serious | No | No | No | High |
| Man, 2018 ^26^ | Hepatocellular carcinoma | OS | No serious | No serious | Serious limitation ^b^ | No serious | Serious limitation ^f^ | No | No | No | Very low |
| Man, 2018 ^26^ | Hepatocellular carcinoma | DFS | No serious | No serious | Serious limitation ^b^ | No serious | Serious limitation ^f^ | No | No | No | Very low |
| Man, 2018 ^26^ | Hepatocellular carcinoma | RFS | No serious | No serious | Serious limitation ^c^ | No serious | No serious | No | No | No | Moderate |
| Pierce, 2024 ^32^ | Colorectal cancer/Gastric Cancer | OS | No serious | No serious | Serious limitation ^c^ | No serious | Serious limitation ^f^ | Yes ^d^ | No | No | Moderate |
| Ren, 2022 ^35^ | Gastrointestinal stromal tumours | RFS (UVA) | No serious | No serious | No serious | No serious | No serious | Yes ^d^ | No | No | High |
| Ren, 2022 ^35^ | Gastrointestinal stromal tumours | RFS (MVA) | No serious | No serious | No serious | No serious | No serious | No | No | No | High |
| Sun, 2019 ^39^ | Colorectal cancer | OS | No serious | No serious | Serious limitation ^b^ | No serious | Serious limitation ^f^ | No | No | No | Very low |
| Sun, 2019 ^39^ | Colorectal cancer | OS | No serious | No serious | Serious limitation ^c^ | No serious | No serious | No | No | No | Moderate |
| Sun, 2019 ^39^ | Colorectal cancer | OS | No serious | No serious | Serious limitation ^c^ | No serious | Serious limitation ^f^ | No | No | No | Low |
| Sun, 2019 ^39^ | Colorectal cancer | OS | No serious | No serious | Serious limitation ^c^ | No serious | Serious limitation ^f^ | No | No | No | Low |
| Sun, 2019 ^39^ | Colorectal cancer | OS | No serious | No serious | Serious limitation ^c^ | No serious | No serious | No | No | No | Moderate |
| Sun, 2019 ^39^ | Colorectal cancer | Serious postoperative complications | No serious | No serious | No serious | No serious | Serious limitation ^f^ | Yes ^d^ | No | No | High |
| Xue, 2019 ^51^ | Esophageal Cancer | OS | No serious | No serious | Serious limitation ^c^ | No serious | Serious limitation ^f^ | No | No | No | Low |
| Xue, 2019 ^51^ | Esophageal Cancer | OS（MVA） | No serious | No serious | Serious limitation ^c^ | No serious | Serious limitation ^f^ | No | No | No | Low |
| Yang, 2016 (a) ^53^ | Gastric cancer | OS | No serious | No serious | No serious | No serious | No serious | No | No | No | High |
| Yang, 2016 (b) ^54^ | Colorectal cancer | OS | No serious | No serious | Serious limitation ^c^ | No serious | Serious limitation ^f^ | No | No | No | Low |
| Yang, 2016 (b) ^54^ | Colorectal cancer | CSS | No serious | No serious | No serious | No serious | No serious | No | No | No | High |
| Yu, 2024 ^55^ | Pancreatic cancer | OS | No serious | No serious | Serious limitation ^b^ | No serious | No serious | No | No | No | Low |
| Zeng, 2025 ^56^ | Biliary tract cancer | OS | No serious | No serious | Serious limitation ^c^ | No serious | No serious | No | No | No | Moderate |
| Zeng, 2025 ^56^ | Cholangiocarcinoma | OS | No serious | No serious | No serious | No serious | No serious | Yes ^d^ | No | No | High |
| Zeng, 2025 ^56^ | Biliary tract cancers | OS | No serious | No serious | No serious | No serious | No serious | No | No | No | High |
| Zeng, 2025 ^56^ | Gallbladder cancer | OS | No serious | No serious | Serious limitation ^b^ | No serious | No serious | Yes ^d^ | No | No | Moderate |
| Zeng, 2025 ^56^ | Gallbladder cancer | OS | No serious | No serious | Serious limitation ^c^ | No serious | No serious | No | No | No | Moderate |
| Zeng, 2025 ^56^ | Biliary tract cancer | DFS | No serious | No serious | No serious | No serious | No serious | No | No | No | High |
| Zhang, 2023 ^58^ | Gastric cancer | OS | No serious | No serious | No serious | No serious | No serious | Yes ^d^ | No | No | High |
| Zhang, 2023 ^58^ | Hepatocellular carcinoma | OS | No serious | No serious | No serious | No serious | No serious | No | No | No | High |
| Zhang, 2023 ^58^ | Esophageal Cancer | OS | No serious | No serious | No serious | Serious limitation ^e^ | No serious | No | No | No | Moderate |
| Zhang, 2023 ^58^ | Gastrointestinal cancer | OS | No serious | No serious | No serious | No serious | Serious limitation ^f^ | No | No | No | Moderate |
| Zhang, 2023 ^58^ | Gastrointestinal cancer | OS | No serious | No serious | Serious limitation ^c^ | No serious | No serious | No | No | No | Moderate |
| Zhang, 2023 ^58^ | Gastrointestinal cancer | OS | No serious | No serious | No serious | No serious | Serious limitation ^f^ | No | No | No | Moderate |
| Zhang, 2023 ^58^ | Gastrointestinal cancer | OS | No serious | No serious | Serious limitation ^c^ | Serious limitation ^e^ | No serious | No | No | No | Low |
| Zhang, 2023 ^58^ | Gastrointestinal cancer | OS (UVA) | No serious | No serious | No serious | No serious | No serious | No | No | No | High |
| Zhang, 2023 ^58^ | Gastrointestinal cancer | OS (MVA) | No serious | No serious | No serious | No serious | Serious limitation ^f^ | No | No | No | Moderate |
| Zhang, 2023 ^58^ | Gastric cancer | PFS | No serious | No serious | No serious | Serious limitation ^e^ | No serious | No | No | No | Moderate |
| Zhang, 2023 ^58^ | Esophageal Cancer | PFS | No serious | No serious | No serious | Serious limitation ^e^ | No serious | No | No | No | Moderate |
| Zhang, 2023 ^58^ | Gastrointestinal cancer | PFS | No serious | No serious | No serious | No serious | Serious limitation ^f^ | No | No | No | Moderate |
| Zhang, 2023 ^58^ | Gastrointestinal cancer | PFS | No serious | No serious | No serious | No serious | Serious limitation ^f^ | No | No | No | Moderate |
| Zhang, 2023 ^58^ | Gastrointestinal cancer | PFS (MVA) | No serious | No serious | No serious | No serious | Serious limitation ^f^ | No | No | No | Moderate |
| Zhang, 2023 ^58^ | Gastrointestinal cancer | PFS (UVA) | No serious | No serious | No serious | Serious limitation ^e^ | No serious | No | No | No | Moderate |
| Zhang, 2024 (a) ^59^ | Hepatocellular carcinoma | OS | No serious | No serious | No serious | No serious | Serious limitation ^f^ | No | No | No | Moderate |
| Zhang, 2024 (a) ^59^ | Hepatocellular carcinoma | RFS | No serious | No serious | Serious limitation ^c^ | No serious | No serious | No | No | No | Moderate |
| Zhao, 2016 ^61^ | Gastric cancer, colorectal cancer, hepatocellular carcinoma, | OS | No serious | No serious | Serious limitation ^c^ | No serious | Serious limitation ^f^ | No | No | No | Low |
| Zhao, 2016 ^61^ | Gastric cancer, colorectal cancer, hepatocellular carcinoma, | OS | No serious | No serious | No serious | No serious | Serious limitation ^f^ | No | No | No | Moderate |
| Zhao, 2016 ^61^ | Gastric cancer, colorectal cancer, hepatocellular carcinoma, | OS | Serious limitation ^a^ | No serious | No serious | No serious | No serious | Yes ^d^ | No | No | High |
| Zhao, 2016 ^61^ | Gastric cancer, colorectal cancer, hepatocellular carcinoma, | OS | No serious | No serious | No serious | No serious | Serious limitation ^f^ | Yes ^d^ | No | No | High |
| Zhao, 2016 ^61^ | Colorectal carcinoma | OS | No serious | No serious | Serious limitation ^c^ | No serious | Serious limitation ^f^ | No | No | No | Low |
| Zhao, 2016 ^61^ | Hepatocellular carcinoma | OS | No serious | No serious | Serious limitation ^c^ | No serious | No serious | No | No | No | Moderate |
| Zhao, 2016 ^61^ | Pancreatic carcinoma | OS | No serious | No serious | No serious | No serious | Serious limitation ^f^ | No | No | No | Moderate |
| Zhao, 2016 ^61^ | Gastric cancer, colorectal cancer, hepatocellular carcinoma, | DFS | No serious | No serious | Serious limitation ^c^ | No serious | No serious | No | No | No | Moderate |
| Zhao, 2016 ^61^ | Gastric cancer, colorectal cancer, hepatocellular carcinoma, | Post-operative complications | No serious | No serious | No serious | No serious | Serious limitation ^f^ | Yes ^d^ | No | No | High |
| Zhao, 2016 ^61^ | Gastric cancer, colorectal cancer, hepatocellular carcinoma, | Post-operative complications/DFS/CSS | No serious | No serious | Serious limitation ^c^ | No serious | Serious limitation ^f^ | No | No | No | Low |
| Zhao, 2022 ^63^ | Pancreatic Cancer | OS | No serious | No serious | No serious | No serious | No serious | No | No | No | High |
| **Respiratory system cancers** | | | | | | | | | | | |
| Jiang, 2020 ^8^ | Small cell lung cancer | OS | No serious | No serious | Serious limitation ^c^ | No serious | No serious | No | No | No | Moderate |
| Jiang, 2020 ^8^ | Small cell lung cancer | PFS | No serious | No serious | Serious limitation ^c^ | No serious | No serious | No | No | No | Moderate |
| Li, 2018 (b) ^14^ | Lung cancer | OS | No serious | No serious | Serious limitation ^c^ | No serious | Serious limitation ^f^ | No | No | No | Low |
| Li, 2018 (b) ^14^ | Non-small cell lung cancer | OS | No serious | No serious | Serious limitation ^c^ | No serious | Serious limitation ^f^ | No | No | No | Low |
| Shao, 2022 ^36^ | Lung cancer | OS | No serious | No serious | Serious limitation ^c^ | No serious | No serious | Yes ^d^ | No | No | High |
| Shao, 2022 ^36^ | Lung cancer | PFS | No serious | No serious | No serious | No serious | No serious | No | No | No | High |
| Wang, 2018 ^46^ | Non-small cell lung cancer | OS | No serious | No serious | Serious limitation ^b^ | No serious | Serious limitation ^f^ | No | No | No | Very low |
| Wang, 2018 ^46^ | Small cell lung cancer | OS | No serious | No serious | Serious limitation ^b^ | No serious | No serious | No | No | No | Low |
| Wang, 2018 ^46^ | Non-small cell lung cancer | DFS/RFS | No serious | No serious | Serious limitation ^b^ | No serious | No serious | No | No | No | Low |
| Wang, 2018 ^46^ | Non-small cell lung cancer | RFS | No serious | No serious | No serious | No serious | No serious | No | No | No | High |
| Wang, 2024 ^48^ | Lung cancer | OS | No serious | No serious | Serious limitation ^c^ | No serious | No serious | Yes ^d^ | No | No | High |
| Wang, 2024 ^48^ | Lung cancer | PFS | No serious | No serious | Serious limitation ^c^ | No serious | No serious | No | No | No | Moderate |
| Xia, 2023 ^49^ | Non-small cell lung cancer | OS | No serious | No serious | Serious limitation ^b^ | No serious | Serious limitation ^f^ | No | No | No | Very low |
| Xia, 2023 ^49^ | Non-small cell lung cancer | PFS | No serious | No serious | Serious limitation ^b^ | No serious | No serious | No | No | No | Low |
| Yan, 2023 ^52^ | Lung cancer | OS | No serious | No serious | Serious limitation ^c^ | No serious | No serious | Yes ^d^ | No | No | High |
| Yan, 2023 ^52^ | Lung cancer | PFS | No serious | No serious | No serious | No serious | No serious | No | No | No | High |
| Zhang, 2021 ^57^ | Lung cancer | OS | Serious limitation ^a^ | No serious | Serious limitation ^b^ | No serious | No serious | No | No | No | Very low |
| Zhang, 2021 ^57^ | Small lung cancer | OS | Serious limitation ^a^ | No serious | Serious limitation ^b^ | No serious | No serious | No | No | No | Very low |
| Zhang, 2021 ^57^ | Non-small lung cancer | OS | Serious limitation ^a^ | No serious | Serious limitation ^b^ | No serious | No serious | No | No | No | Very low |
| Zhang, 2021 ^57^ | Lung cancer | OS | Serious limitation ^a^ | No serious | Serious limitation ^b^ | No serious | No serious | No | No | No | Very low |
| Zhang, 2021 ^57^ | Lung cancer | PFS | Serious limitation ^a^ | No serious | Serious limitation ^b^ | No serious | No serious | No | No | No | Very low |
| Zhang, 2021 ^57^ | Non-small cell lung cancer | PFS | Serious limitation ^a^ | No serious | Serious limitation ^b^ | No serious | No serious | No | No | No | Very low |
| **Urinary system cancers** | | | | | | | | | | | |
| Jiao, 2023 ^10^ | Bladder Cancer | OS | No serious | No serious | No serious | No serious | No serious | No | No | No | High |
| Jiao, 2023 ^10^ | Bladder Cancer | RFS | No serious | No serious | Serious limitation ^b^ | No serious | No serious | No | No | No | Low |
| Kim, 2021 ^12^ | Renal cell carcinoma | RFS/DFS | No serious | No serious | No serious | No serious | No serious | No | No | No | High |
| Kim, 2021 ^12^ | Renal cell carcinoma | OS/CSS | No serious | No serious | No serious | No serious | Serious limitation ^f^ | No | No | No | Moderate |
| Mao, 2021 ^27^ | Non-metastatic Renal cell cancer | OS | No serious | No serious | Serious limitation ^c^ | No serious | No serious | Yes ^d^ | No | No | High |
| Mao, 2021 ^27^ | Metastatic Renal cell cancer | OS | No serious | No serious | Serious limitation ^c^ | No serious | No serious | Yes ^d^ | No | No | High |
| Mao, 2021 ^27^ | Renal cell carcinoma | OS | No serious | No serious | Serious limitation ^c^ | No serious | No serious | Yes ^d^ | No | No | High |
| Mao, 2021 ^27^ | Clear cell renal cell carcinoma/Non-clear cell renal cell carcinoma | OS | No serious | No serious | Serious limitation ^c^ | No serious | No serious | Yes ^d^ | No | No | High |
| Mao, 2021 ^27^ | Renal cell carcinoma | OS | No serious | No serious | No serious | No serious | No serious | No | No | No | High |
| Mao, 2021 ^27^ | Renal cell carcinoma | OS | No serious | No serious | Serious limitation ^c^ | No serious | No serious | Yes ^d^ | No | No | High |
| Mao, 2021 ^27^ | Renal cell carcinoma | OS | No serious | No serious | Serious limitation ^c^ | No serious | No serious | Yes ^d^ | No | No | High |
| Mao, 2021 ^27^ | Renal cell carcinoma | CSS | No serious | No serious | Serious limitation ^c^ | No serious | No serious | Yes ^d^ | No | No | High |
| Mao, 2021 ^27^ | Renal cell carcinoma | CSS | No serious | No serious | No serious | No serious | No serious | Yes ^d^ | No | No | High |
| Mao, 2021 ^27^ | Renal cell carcinoma | CSS | No serious | No serious | Serious limitation ^c^ | No serious | No serious | Yes ^d^ | No | No | High |
| Mao, 2021 ^27^ | Renal Cell Carcinoma | PFS/DFS/RFS | No serious | No serious | No serious | No serious | No serious | Yes ^d^ | No | No | High |
| Mao, 2021 ^27^ | Renal Cell Carcinoma | PFS/DFS/RFS | No serious | No serious | No serious | No serious | Serious limitation ^f^ | No | No | No | Moderate |
| Mao, 2021 ^27^ | Clear cell renal cell carcinoma/Non-clear cell renal cell carcinoma | PFS/DFS/RFS | No serious | No serious | No serious | No serious | Serious limitation ^f^ | No | No | No | Moderate |
| Mao, 2021 ^27^ | Renal cell carcinoma | PFS/DFS/RFS | No serious | No serious | No serious | No serious | No serious | Yes ^d^ | No | No | High |
| Mao, 2021 ^27^ | Renal cell carcinoma | PFS/DFS/RFS | No serious | No serious | No serious | No serious | No serious | No | No | No | High |
| Mao, 2021 ^27^ | Renal cell carcinoma | PFS/DFS/RFS | No serious | No serious | No serious | No serious | No serious | No | No | No | High |
| Meng, 2022 ^29^ | Upper tract urothelial carcinoma | OS | No serious | No serious | No serious | No serious | Serious limitation ^f^ | No | No | No | Moderate |
| Meng, 2022 ^29^ | Upper tract urothelial carcinoma | DFS/RFS/PFS | No serious | No serious | No serious | No serious | No serious | No | No | No | High |
| Meng, 2022 ^29^ | Upper tract urothelial carcinoma | CSS/DSS | No serious | No serious | No serious | No serious | No serious | No | No | No | High |
| Qi, 2018 ^34^ | Urinary cancers | OS | No serious | No serious | No serious | No serious | No serious | No | No | No | High |
| Qi, 2018 ^34^ | Urinary cancers | OS | No serious | No serious | No serious | No serious | No serious | No | No | No | High |
| Qi, 2018 ^34^ | Urinary cancers | OS | No serious | No serious | No serious | No serious | No serious | No | No | No | High |
| Qi, 2018 ^34^ | Renal cell cancer | OS | No serious | No serious | No serious | No serious | Serious limitation ^f^ | No | No | No | Moderate |
| Qi, 2018 ^34^ | Urinary cancers | CSS/DSS | No serious | No serious | No serious | No serious | No serious | No | No | No | High |
| Qi, 2018 ^34^ | Urinary cancers | DFS/RFS /PFS | No serious | No serious | No serious | No serious | No serious | No | No | No | High |
| Qi, 2018 ^34^ | Urinary cancers | DFS/RFS /PFS | No serious | No serious | No serious | No serious | No serious | Yes ^d^ | No | No | High |
| Qi, 2018 ^34^ | Urinary cancers | DFS/RFS /PFS | No serious | No serious | No serious | No serious | No serious | No | No | No | High |
| Qi, 2018 ^34^ | Renal cell cancer | DFS/RFS/PFS | No serious | No serious | No serious | No serious | Serious limitation ^f^ | No | No | No | Moderate |
| Sun, 2025 ^40^ | Bladder cancer | OS | No serious | No serious | No serious | No serious | No serious | No | No | No | High |
| Sun, 2025 ^40^ | Bladder cancer | RFS | No serious | No serious | Serious limitation ^c^ | No serious | No serious | No | No | No | Moderate |
| Tang, 2021 ^43^ | Renal cell carcinoma | OS | No serious | No serious | No serious | No serious | No serious | No | No | No | High |
| Tang, 2021 ^43^ | Renal cell carcinoma | RFS | No serious | No serious | No serious | No serious | Serious limitation ^f^ | No | No | No | Moderate |
| Zhao, 2019 ^62^ | Urologic tumors | OS | No serious | No serious | No serious | No serious | Serious limitation ^f^ | No | No | No | Moderate |
| Zhao, 2019 ^62^ | Urologic tumors | OS | No serious | No serious | No serious | No serious | No serious | No | No | No | High |
| Zhao, 2019 ^62^ | Urologic tumors | OS | No serious | No serious | No serious | No serious | Serious limitation ^f^ | No | No | No | Moderate |
| **Reproductive system cancers** | | | | | | | | | | | |
| Cao, 2024 ^2^ | Cervical cancer | OS | No serious | No serious | Serious limitation ^b^ | No serious | No serious | No | No | No | Low |
| Cao, 2024 ^2^ | Cervical cancer | OS | No serious | No serious | Serious limitation ^b^ | No serious | No serious | No | No | No | Low |
| Cao, 2024 ^2^ | Cervical cancer | OS | No serious | No serious | Serious limitation ^b^ | No serious | No serious | No | No | No | Low |
| Cao, 2024 ^2^ | Cervical cancer | OS | No serious | No serious | No serious | No serious | Serious limitation ^f^ | Yes ^d^ | No | No | High |
| Cao, 2024 ^2^ | Cervical cancer | OS | No serious | No serious | Serious limitation ^b^ | No serious | No serious | No | No | No | Low |
| Cao, 2024 ^2^ | Cervical cancer | OS | No serious | No serious | Serious limitation ^b^ | No serious | No serious | No | No | No | Low |
| Cao, 2024 ^2^ | Cervical cancer | OS | No serious | No serious | Serious limitation ^b^ | No serious | No serious | No | No | No | Low |
| Cao, 2024 ^2^ | Cervical cancer | OS | No serious | No serious | Serious limitation ^b^ | No serious | No serious | No | No | No | Low |
| Cao, 2024 ^2^ | Cervical cancer | OS | No serious | No serious | Serious limitation ^b^ | No serious | Serious limitation ^f^ | No | No | No | Very low |
| Cao, 2024 ^2^ | Cervical cancer | OS | No serious | No serious | Serious limitation ^b^ | No serious | No serious | No | No | No | Low |
| Cao, 2024 ^2^ | Cervical cancer | OS | No serious | No serious | Serious limitation ^b^ | No serious | No serious | No | No | No | Low |
| Cao, 2024 ^2^ | Cervical cancer | OS | No serious | No serious | No serious | No serious | No serious | Yes ^d^ | No | No | High |
| Cao, 2024 ^2^ | Cervical cancer | OS | No serious | No serious | Serious limitation ^b^ | No serious | No serious | No | No | No | Low |
| Cao, 2024 ^2^ | Cervical cancer | OS | No serious | No serious | Serious limitation ^b^ | No serious | No serious | No | No | No | Low |
| Cao, 2024 ^2^ | Cervical cancer | OS | No serious | No serious | Serious limitation ^b^ | No serious | No serious | No | No | No | Low |
| Cao, 2024 ^2^ | Cervical cancer | OS | No serious | No serious | Serious limitation ^b^ | No serious | No serious | No | No | No | Low |
| Cao, 2024 ^2^ | Cervical cancer | OS | No serious | No serious | No serious | No serious | Serious limitation ^f^ | No | No | No | Moderate |
| Cao, 2024 ^2^ | Cervical cancer | OS | No serious | No serious | No serious | No serious | No serious | No | No | No | High |
| Cao, 2024 ^2^ | Cervical cancer | OS | No serious | No serious | Serious limitation ^b^ | No serious | Serious limitation ^f^ | Yes ^d^ | No | No | Low |
| Cao, 2024 ^2^ | Cervical cancer | OS | No serious | No serious | No serious | No serious | No serious | No | No | No | High |
| Cao, 2024 ^2^ | Cervical cancer | PFS | No serious | No serious | Serious limitation ^b^ | No serious | No serious | No | No | No | Low |
| Cao, 2024 ^2^ | Cervical cancer | PFS | No serious | No serious | Serious limitation ^b^ | No serious | No serious | No | No | No | Low |
| Cao, 2024 ^2^ | Cervical cancer | PFS | No serious | No serious | Serious limitation ^b^ | No serious | Serious limitation ^f^ | No | No | No | Very low |
| Cao, 2024 ^2^ | Cervical cancer | PFS | No serious | No serious | Serious limitation ^b^ | No serious | No serious | No | No | No | Low |
| Cao, 2024 ^2^ | Cervical cancer | PFS | No serious | No serious | Serious limitation ^b^ | No serious | No serious | No | No | No | Low |
| Cao, 2024 ^2^ | Cervical cancer | PFS | No serious | No serious | Serious limitation ^b^ | No serious | Serious limitation ^f^ | No | No | No | Very low |
| Cao, 2024 ^2^ | Cervical cancer | PFS | No serious | No serious | Serious limitation ^b^ | No serious | No serious | No | No | No | Low |
| Li, 2024 ^19^ | Ovarian cancer/cervical cancer patients/endometrial cancer | OS | No serious | No serious | Serious limitation ^b^ | No serious | Serious limitation ^f^ | No | No | No | Very low |
| Li, 2024 ^19^ | Ovarian cancer/cervical cancer patients/endometrial cancer | PFS | No serious | No serious | Serious limitation ^b^ | No serious | No serious | No | No | No | Low |
| Li, 2024 ^19^ | Ovarian cancer/cervical cancer patients/endometrial cancer | DFS | No serious | No serious | Serious limitation ^b^ | No serious | Serious limitation ^f^ | No | No | No | Very low |
| Mao, 2024 ^28^ | Endometrial cancer | OS | No serious | No serious | Serious limitation ^b^ | No serious | Serious limitation ^f^ | No | No | No | Very low |
| Mao, 2024 ^28^ | Endometrial cancer | PFS | No serious | No serious | Serious limitation ^b^ | No serious | No serious | Yes ^d^ | No | No | Moderate |
| Niu, 2023 ^31^ | Cervical cancer | OS | No serious | No serious | No serious | No serious | No serious | Yes ^d^ | No | No | High |
| Niu, 2023 ^31^ | Cervical cancer | PFS | No serious | No serious | No serious | No serious | Serious limitation ^f^ | Yes ^d^ | No | No | High |
| Tan, 2022 ^41^ | Ovarian Cancer | OS | No serious | No serious | Serious limitation ^b^ | No serious | No serious | No | No | No | Low |
| Tan, 2022 ^41^ | Ovarian Cancer | PFS | No serious | No serious | Serious limitation ^b^ | No serious | Serious limitation ^f^ | No | No | No | Very low |
| Tobing, 2024 ^44^ | Prostate Cancer | OS | No serious | No serious | Serious limitation ^c^ | No serious | Serious limitation ^f^ | No | No | No | Low |
| Tobing, 2024 ^44^ | Prostate Cancer | PFS | No serious | No serious | No serious | No serious | Serious limitation ^f^ | No | No | No | Moderate |
| Wang, 2019 ^47^ | Gynecological cancers | OS (UVA) | No serious | No serious | Serious limitation ^b^ | No serious | Serious limitation ^f^ | Yes ^d^ | No | No | Low |
| Wang, 2019 ^47^ | Gynecological cancers | OS (MVA) | No serious | No serious | Serious limitation ^b^ | No serious | Serious limitation ^f^ | No | No | No | Very low |
| Wang, 2019 ^47^ | Gynecological cancers | PFS (UVA） | No serious | No serious | No serious | No serious | No serious | Yes ^d^ | No | No | High |
| Wang, 2019 ^47^ | Gynecological cancers | PFS (MVA) | No serious | No serious | No serious | No serious | No serious | No | No | No | High |
| Zhang, 2024 (b) ^60^ | Endometrial cancer | OS | No serious | No serious | Serious limitation ^c^ | No serious | Serious limitation ^f^ | Yes ^d^ | No | No | Moderate |
| Zhang, 2024 (b) ^60^ | Endometrial cancer | OS | No serious | No serious | Serious limitation ^c^ | No serious | Serious limitation ^f^ | No | No | No | Low |
| Zhang, 2024 (b) ^60^ | Endometrial cancer | OS | No serious | No serious | Serious limitation ^c^ | No serious | Serious limitation ^f^ | Yes ^d^ | No | No | Moderate |
| Zhang, 2024 (b) ^60^ | Endometrial cancer | OS | No serious | No serious | Serious limitation ^c^ | No serious | No serious | No | No | No | Moderate |
| Zhang, 2024 (b) ^60^ | Endometrial cancer | OS | No serious | No serious | No serious | No serious | Serious limitation ^f^ | No | No | No | Moderate |
| Zhang, 2024 (b) ^60^ | Endometrial cancer | OS | No serious | No serious | No serious | No serious | No serious | Yes ^d^ | No | No | High |
| Zhang, 2024 (b) ^60^ | Endometrial cancer | OS | No serious | No serious | No serious | No serious | No serious | No | No | No | High |
| Zhang, 2024 (b) ^60^ | Endometrial cancer | OS | No serious | No serious | Serious limitation ^b^ | No serious | Serious limitation ^f^ | Yes ^d^ | No | No | Low |
| Zhang, 2024 (b) ^60^ | Endometrial cancer | OS | No serious | No serious | Serious limitation ^c^ | No serious | Serious limitation ^f^ | Yes ^d^ | No | No | Moderate |
| Zhang, 2024 (b) ^60^ | Endometrial cancer | OS | No serious | No serious | Serious limitation ^c^ | No serious | No serious | No | No | No | Moderate |
| Zhang, 2024 (b) ^60^ | Endometrial cancer | PFS | No serious | No serious | Serious limitation ^b^ | No serious | Serious limitation ^f^ | Yes ^d^ | No | No | Low |
| Zhang, 2024 (b) ^60^ | Endometrial cancer | PFS | No serious | No serious | Serious limitation ^b^ | No serious | No serious | Yes ^d^ | No | No | Moderate |
| Zhang, 2024 (b) ^60^ | Endometrial cancer | PFS | No serious | No serious | Serious limitation ^c^ | No serious | No serious | Yes ^d^ | No | No | High |
| Zhang, 2024 (b) ^60^ | Endometrial cancer | PFS | No serious | No serious | Serious limitation ^c^ | No serious | No serious | Yes ^d^ | No | No | High |
| Zhang, 2024 (b) ^60^ | Endometrial cancer | PFS | No serious | No serious | Serious limitation ^b^ | No serious | No serious | Yes ^d^ | No | No | Moderate |
| Zhang, 2024 (b) ^60^ | Endometrial cancer | PFS | No serious | No serious | Serious limitation ^b^ | No serious | No serious | Yes ^d^ | No | No | Moderate |
| Zhang, 2024 (b) ^60^ | Endometrial cancer | PFS | No serious | No serious | Serious limitation ^b^ | No serious | No serious | Yes ^d^ | No | No | Moderate |
| Zheng, 2023 ^64^ | Prostate cancer | OS | No serious | No serious | Serious limitation ^c^ | No serious | Serious limitation ^f^ | Yes ^d^ | No | No | Moderate |
| Zheng, 2023 ^64^ | Prostate cancer | OS | No serious | No serious | No serious | Serious limitation ^e^ | No serious | Yes ^d^ | No | No | High |
| Zheng, 2023 ^64^ | Prostate cancer | OS | No serious | No serious | Serious limitation ^c^ | No serious | No serious | No | No | No | Moderate |
| Zheng, 2023 ^64^ | Prostate cancer | PFS | No serious | No serious | Serious limitation ^c^ | No serious | No serious | Yes ^d^ | No | No | High |
| Zheng, 2023 ^64^ | Prostate cancer | PFS | No serious | No serious | No serious | Serious limitation ^e^ | No serious | Yes ^d^ | No | No | High |
| Zheng, 2023 ^64^ | Prostate cancer | PFS | No serious | No serious | No serious | No serious | No serious | No | No | No | High |
| **Head and neck cancers** | | | | | | | | | | | |
| Dai, 2023 ^3^ | Oral Cancer | OS | No serious | No serious | Serious limitation ^b^ | No serious | Serious limitation ^f^ | Yes ^d^ | No | No | Low |
| Dai, 2023 ^3^ | Oral Cancer | DFS | No serious | No serious | No serious | No serious | Serious limitation ^f^ | No | No | No | Moderate |
| Luan, 2021 ^24^ | Head and neck cancer | OS | No serious | No serious | No serious | No serious | Serious limitation ^f^ | No | No | No | Moderate |
| Luan, 2021 ^24^ | Oral cavity | OS | No serious | No serious | Serious limitation ^c^ | No serious | Serious limitation ^f^ | No | No | No | Low |
| Luan, 2021 ^24^ | Nasopharynx | OS | No serious | No serious | No serious | No serious | No serious | No | No | No | High |
| Luan, 2021 ^24^ | Head and neck cancer | PFS | No serious | No serious | No serious | No serious | No serious | No | No | No | High |
| Luan, 2021 ^24^ | Head and neck cancer | DMFS | No serious | No serious | No serious | No serious | No serious | Yes ^d^ | No | No | High |
| Luan, 2021 ^24^ | Head and neck cancer | DFS | No serious | No serious | Serious limitation ^b^ | No serious | No serious | No | No | No | Low |
| Luan, 2021 ^24^ | Head and neck cancer | DSS | No serious | No serious | No serious | No serious | Serious limitation ^f^ | Yes ^d^ | No | No | High |
| Shi, 2021 ^37^ | Head and neck cancer | OS | No serious | No serious | No serious | No serious | Serious limitation ^f^ | No | No | No | Moderate |
| Shi, 2021 ^37^ | Head and neck cancer | PFS | No serious | No serious | No serious | No serious | No serious | No | No | No | High |
| Shi, 2021 ^37^ | Head and neck cancer | Distant metastasis-free survival | No serious | No serious | No serious | No serious | No serious | No | No | No | High |
| Tang, 2020 ^42^ | Nasopharyngeal carcinoma | OS (UVA) | No serious | No serious | No serious | No serious | No serious | Yes ^d^ | No | No | High |
| Tang, 2020 ^42^ | Nasopharyngeal carcinoma | OS (MVA) | No serious | No serious | No serious | No serious | No serious | No | No | No | High |
| Tang, 2020 ^42^ | Nasopharyngeal carcinoma | PFS (UVA) | No serious | No serious | Serious limitation ^c^ | No serious | No serious | Yes ^d^ | No | No | High |
| Tang, 2020 ^42^ | Nasopharyngeal carcinoma | PFS (MVA) | No serious | No serious | No serious | No serious | No serious | No | No | No | High |
| Tang, 2020 ^42^ | Nasopharyngeal carcinoma | DMFS (UVA) | No serious | No serious | No serious | No serious | No serious | Yes ^d^ | No | No | High |
| Tang, 2020 ^42^ | Nasopharyngeal carcinoma | DMFS (MVA) | No serious | No serious | No serious | No serious | No serious | Yes ^d^ | No | No | High |
| Tu, 2020 ^45^ | Nasopharyngeal carcinoma | OS | No serious | No serious | No serious | No serious | Serious limitation ^f^ | No | No | No | Moderate |
| Tu, 2020 ^45^ | Nasopharyngeal carcinoma | PFS | No serious | No serious | No serious | No serious | No serious | No | No | No | High |
| Tu, 2020 ^45^ | Nasopharyngeal carcinoma | DMFS | No serious | No serious | No serious | No serious | No serious | Yes ^d^ | No | No | High |
| Tu, 2020 ^45^ | Nasopharyngeal carcinoma | Locoregional RFS | No serious | No serious | No serious | No serious | No serious | No | No | No | High |
| **Hematologic system cancers** | | | | | | | | | | | |
| Luan, 2020 ^23^ | Diffuse large b-cell lymphoma | OS | No serious | No serious | No serious | No serious | No serious | Yes ^d^ | No | No | High |
| Luan, 2020 ^23^ | Diffuse large b-cell lymphoma | PFS | No serious | No serious | No serious | No serious | No serious | No | No | No | High |
| **Nervous system cancers** | | | | | | | | | | | |
| Hung, 2023 ^7^ | Gliomas | OS | No serious | No serious | No serious | No serious | No serious | No | No | No | High |
| Hung, 2023 ^7^ | Gliomas | OS（MVA） | No serious | No serious | No serious | No serious | No serious | No | No | No | High |
| Hung, 2023 ^7^ | Gliomas | OS (UVA) | No serious | No serious | No serious | No serious | No serious | No | No | No | High |
| Hung, 2023 ^7^ | Gliomas | PFS | No serious | No serious | No serious | No serious | No serious | No | No | No | High |
| Liu, 2020 ^21^ | Glioblastoma multiform | OS | No serious | No serious | Serious limitation ^c^ | No serious | No serious | No | No | No | Moderate |
| Liu, 2020 ^21^ | Gliomas | OS | No serious | No serious | Serious limitation ^b^ | No serious | No serious | No | No | No | Low |
| Liu, 2020 ^21^ | Gliomas | OS（UVA） | No serious | No serious | Serious limitation ^b^ | No serious | No serious | No | No | No | Low |
| Liu, 2020 ^21^ | Gliomas | OS（MVA） | No serious | No serious | No serious | No serious | No serious | No | No | No | High |
| **Breast cancers** | | | | | | | | | | | |
| Prasetiyo, 2023 ^33^ | Breast cancer | OS | No serious | No serious | No serious | No serious | No serious | Yes ^d^ | No | No | High |
| Prasetiyo, 2023 ^33^ | Breast cancer | DFS | No serious | No serious | Serious limitation ^b^ | No serious | No serious | No | No | No | Low |
| **Other cancers** | | | | | | | | | | | |
| Bullock, 2020 ^1^ | Cancer | OS | No serious | No serious | Serious limitation ^c^ | No serious | No serious | No | No | No | Moderate |
| Li, 2022 (b) ^18^ | Advanced cancers | OS | No serious | No serious | No serious | No serious | No serious | Yes ^d^ | No | No | High |
| Li, 2022 (b) ^18^ | Advanced cancers | PFS | No serious | No serious | No serious | No serious | No serious | No | No | No | High |
| Ni, 2022 ^30^ | Cancer | OS | No serious | No serious | Serious limitation ^b^ | No serious | No serious | Yes ^d^ | No | No | Moderate |
| Ni, 2022 ^30^ | Cancer | OS | No serious | No serious | Serious limitation ^c^ | No serious | No serious | Yes ^d^ | No | No | High |
| Ni, 2022 ^30^ | Cancer | OS (MVA) | No serious | No serious | Serious limitation ^c^ | No serious | No serious | Yes ^d^ | No | No | High |
| Ni, 2022 ^30^ | Cancer | OS (UVA) | No serious | No serious | No serious | Serious limitation ^e^ | No serious | Yes ^d^ | No | No | High |
| Ni, 2022 ^30^ | Cancer | OS | No serious | No serious | Serious limitation ^c^ | No serious | No serious | Yes ^d^ | No | No | High |
| Ni, 2022 ^30^ | Cancer | PFS | No serious | No serious | No serious | Serious limitation ^e^ | No serious | No | No | No | Moderate |
| Ni, 2022 ^30^ | Cancer | PFS | No serious | No serious | Serious limitation ^c^ | No serious | No serious | No | No | No | Moderate |
| Ni, 2022 ^30^ | Cancer | PFS (MVA) | No serious | No serious | Serious limitation ^c^ | No serious | No serious | No | No | No | Moderate |
| Ni, 2022 ^30^ | Cancer | PFS (UVA) | No serious | No serious | No serious | Serious limitation ^e^ | No serious | No | No | No | Moderate |
| Ni, 2022 ^30^ | Cancer | PFS | No serious | No serious | No serious | No serious | No serious | No | No | No | High |
| Ni, 2022 ^30^ | Cancer | PFS | No serious | No serious | No serious | No serious | No serious | No | No | No | High |
| Ni, 2022 ^30^ | Cancer | Disease control rate | No serious | No serious | No serious | No serious | No serious | Yes ^d^ | No | No | High |
| Ni, 2022 ^30^ | Cancer | Objective response rate | No serious | No serious | Serious limitation ^c^ | No serious | No serious | Yes ^d^ | No | No | High |
| Ni, 2022 ^30^ | Lung cancer (subgroup) | OS | No serious | No serious | Serious limitation ^c^ | No serious | No serious | Yes ^d^ | No | No | High |
| Ni, 2022 ^30^ | Lung cancer (subgroup) | PFS | No serious | No serious | No serious | No serious | No serious | No | No | No | High |
| Sun, 2014 ^38^ | Colorectal cancer (subgroup) | OS | No serious | No serious | No serious | No serious | Serious limitation ^f^ | No | No | No | Moderate |
| Sun, 2014 ^38^ | Cancer | OS | No serious | No serious | No serious | No serious | Serious limitation ^f^ | No | No | No | Moderate |
| Sun, 2014 ^38^ | Cancer | OS | No serious | No serious | No serious | No serious | No serious | No | No | No | High |
| Sun, 2014 ^38^ | Cancer | OS | No serious | No serious | No serious | No serious | No serious | Yes ^d^ | No | No | High |
| Sun, 2014 ^38^ | Cancer | OS | No serious | No serious | No serious | No serious | No serious | No | No | No | High |
| Xu, 2023 ^50^ | Cancer | OS | No serious | No serious | Serious limitation ^c^ | No serious | No serious | Yes ^d^ | No | No | High |
| Xu, 2023 ^50^ | Cancer | PFS | No serious | No serious | No serious | No serious | No serious | No | No | No | High |
| Xu, 2023 ^50^ | Cancer | Disease control rate | No serious | No serious | No serious | No serious | No serious | No | No | No | High |

CSS, cancer-specific survival; DFS, disease-free survival; DMFS, distant metastasis-free survival; MVA, multivariate analyses; OS, overall survival; PFS, progression-free survival; RFS, recurrence-free survival; UVA, univariate analyses.

^a^ More than half of the studies with high risk of bias.

^b^ Moderate heterogeneity is seen between studies (*I^2^* ＞ 75%).

^c^ High heterogeneity is seen between studies (50% < *I^2^* ≤ 75%).

^d^ Large magnitude of effect (OR/RR/HR > 2 or < 0.5).

^e^ Sample size < 400.

^f^ Publication bias were detected (Egger’s *P* value < 0.05, Begg's *P* value < 0.05, or funnel plot is not symmetry).

**Supplementary table 6. Summary of sensitivity analysis after excluding small sample size (****25th percentile) studies: significant associations in high-certainty evidence from more than ten studies**

| **Author, year, ref** | **Cancer site** | **Outcomes** | **Level of comparison** | **Effect metric** | **Primary analysis** | | | **Sensitivity analysis: Excluding studies with small sample size (25th percentile)** | | |
| --- | --- | --- | --- | --- | --- | --- | --- | --- | --- | --- |
|  |  |  |  |  | **No. of studies** | **Summary effects**  **(95% CI)** | **GRADE** | **No. of studies** | **Summary effects**  **(95% CI)** | **GRADE** |
| Hung, 2023 ^7^ | Glioma | OS | High vs. low | HR | 13 | 0.61 (0.52, 0.72) | High | 10 | 0.64 (0.53, 0.78) | High |
| Li, 2018 (a) ^13^ | Gastric cancer | OS | Low vs. high | HR | 15 | 1.81 (1.56, 2.09) | High | 11 | 1.81 (1.55, 2.12) | Moderate |
| Ni, 2022 ^30^ | Advanced-stage cancer | Objective response rate | Low vs. high | HR | 12 | 2.24 (1.57, 3.20) | High | 9 | 2.27 (1.51, 3.41) | High |
| Sun, 2014 ^38^ | Cancer | OS | Low vs. high | OR | 13 | 1.83 (1.58, 2.10) | High | 10 | 1.79 (1.52, 2.10) | High |
| Wang, 2024 ^48^ | Lung cancer | OS | High vs. low | HR | 19 | 0.43 (0.34, 0.54) | High | 16 | 0.43 (0.34, 0.55) | High |
| Xu, 2023 ^50^ | Cancer | OS | Low vs. high | HR | 22 | 2.26 (1.81, 2.82) | High | 17 | 2.29 (1.72, 3.03) | High |
| Xu, 2023 ^50^ | Cancer | PFS | Low vs. high | HR | 19 | 1.75 (1.54, 1.99) | High | 14 | 1.73 (1.48, 2.02) | High |
| Yan, 2023 ^52^ | Advanced lung cancer | OS | Low vs. high | HR | 13 | 2.56 (1.86, 3.54) | High | 10 | 2.45 (1.73, 3.48) | High |
| Zhao, 2022 ^63^ | Pancreatic cancer | OS | Low vs. high | HR | 14 | 1.66 (1.42, 1.94) | High | 10 | 1.70 (1.42, 2.03) | Moderate |

CI, confidence interval; GRADE, Grading of Recommendations, Assessment, Development and Evaluation; HR, hazard ratio; OR, odds ratio; OS, overall survival; PFS, progression-free survival.

**Supplementary table 7. Summary of sensitivity analysis after excluding high risk of bias studies: significant associations in high-certainty evidence with more than ten studies**

| **Author, year, ref** | **Cancer site** | **Outcomes** | **Level of comparison** | **Effect metric** | **Primary analysis** | | | **Sensitivity analysis: Excluding studies with high risk of bias (based on quality assessments).** | | |
| --- | --- | --- | --- | --- | --- | --- | --- | --- | --- | --- |
|  |  |  |  |  | **No. of studies** | **Summary effects**  **(95% CI)** | **GRADE** | **No. of studies** | **Summary effects**  **(95% CI)** | **GRADE** |
| Hung, 2023 ^7^ | Glioma | OS | High vs. low | HR | 13 | 0.61 (0.52, 0.72) | High | 12 | 0.63 (0.53, 0.74) | High |
| Li, 2018 (a) ^13^ | Gastric cancer | OS | Low vs. high | HR | 15 | 1.81 (1.56, 2.09) | High | 7 | 1.84 (1.54, 2.20) | High |
| Ni, 2022 ^30^ | Advanced-stage cancer | Objective response rate | Low vs. high | HR | 12 | 2.24 (1.57, 3.20) | High | N/A – All studies have low risk of bias | | |
| Sun, 2014 ^38^ | Cancer | OS | Low vs. high | OR | 13 | 1.83 (1.58, 2.10) | High | N/A – All studies have low risk of bias | | |
| Wang, 2024 ^48^ | Lung cancer | OS | High vs. low | HR | 19 | 0.43 (0.34, 0.54) | High | N/A – All studies have low risk of bias | | |
| Xu, 2023 ^50^ | Cancer | OS | Low vs. high | HR | 22 | 2.26 (1.81, 2.82) | High | 19 | 2.20 (1.68, 2.88). | High |
| Xu, 2023 ^50^ | Cancer | PFS | Low vs. high | HR | 19 | 1.75 (1.54, 1.99) | High | 17 | 1.78 (1.56, 2.04) | High |
| Yan, 2023 ^52^ | Advanced lung cancer | OS | Low vs. high | HR | 13 | 2.56 (1.86, 3.54) | High | N/A – All studies have low risk of bias | | |
| Zhao, 2022 ^63^ | Pancreatic cancer | OS | Low vs. high | HR | 14 | 1.66 (1.42, 1.94) | High | 7 | 1.61 (1.38, 1.87) | High |

CI, confidence interval; GRADE, Grading of Recommendations, Assessment, Development and Evaluation; HR, hazard ratio; N/A, not applicable; OR, odds ratio; OS, overall survival; PFS, progression-free survival.

**Supplementary table 8. The summary results of meta-analyses excluded due to without quantitative synthesis**

| **Author, Year, Ref** | **No. of studies** | **Cancer site** | **Outcomes** | **Results** |
| --- | --- | --- | --- | --- |
| Feng, 2024 ^65^ | NA | Gastric or gastroesophageal junction adenocarcinoma | OS | A higher the PNI was associated with significantly better survival in patients with gastric cancer. The results provide support for the validity of PNI as a biomarker in predicting the survival of gastric cancer patients. |
| Fiflis, 2024 ^66^ | 16 | Gastric or gastroesophageal junction adenocarcinoma | OS | The 5-year OS of patients in the low-PNI groups ranged between 39% and 70.6%, while in the high-PNI groups, it ranged between 54.9% and 95.8%. In most of the included studies, patients with high preoperative PNI showed statistically significant better OS than the low PNI groups. In multivariate analyses, low PNI was repeatedly recognized as an independent prognostic factor for poor survival. |
| Grimes, 2016 ^67^ | 8 | Renal Cell Carcinoma | OS/RFS/CSS | Only 1 article was identified for PNI. It included 945 patients with various subtypes of RCC and only assessed DFS as its outcome without reporting on the survival of patients. It investigated 2 methods of using PNI, one continuously and the other comparing patients with a score of 48 or less with patients with a score of more than 48. Both were shown to be independent prognostic factors on multivariate analysis. |
| Laszkiewicz, 2024 ^68^ | 94 | Upper Tract Urothelial Carcinoma | OS/CSS/DFS/RFS/PFS | Its prognostic value has been identified in a single meta-analysis by Meng et al.,^29^ who analyzed six retrospective studies including 2324 upper tract urothelial carcinoma patients. The authors revealed that a low pretreatment PNI was associated with worse OS, CSS, DFS, RFS and PFS after RNU. |
| Li, 2024 ^69^ | NA | Small bowel adenocarcinoma | OS/RFS | PNI decreased in patients after SR for SBA (median change: -1.82), particularly in those who underwent Whipple operation or developed postoperative pancreatic fistula. Low postoperative PNI, tumoral lymphovascular invasion, and adjuvant chemotherapy were independent risk factors for OS. Postoperative PNI also significantly predicted recurrence-free survival independent of lymphovascular invasion and adjuvant chemotherapy. |
| Morelli, 2025 ^70^ | 20 | Glioma | OS | UVA showed that patients with a PNI < 44.4 had a median survival of 270 days vs 375 days for those patients with a PNI >= 44.4. At MVA PNI >= 44.4 remained an independent prognostic factor, along with age<60 years and completed adjuvant treatment. |
| Teja, 2025 ^71^ | NA | Pancreatic cancer | OS/RFS | In a multivariable analysis, PNI was associated with RFS and the log-transformed value of PNI was also identified as an independent prognostic marker for OS. |
| Yan, 2021 ^72^ | NA | Cancers | OS/DFS/PFS | The relevant studies indicate that low PNI is an independent prognostic factor for decreasing overall survival in many types of cancers. Disease-free survival and progression-free survival were also associated with PNI in some types of cancer including lung cancer and renal cell carcinoma. |


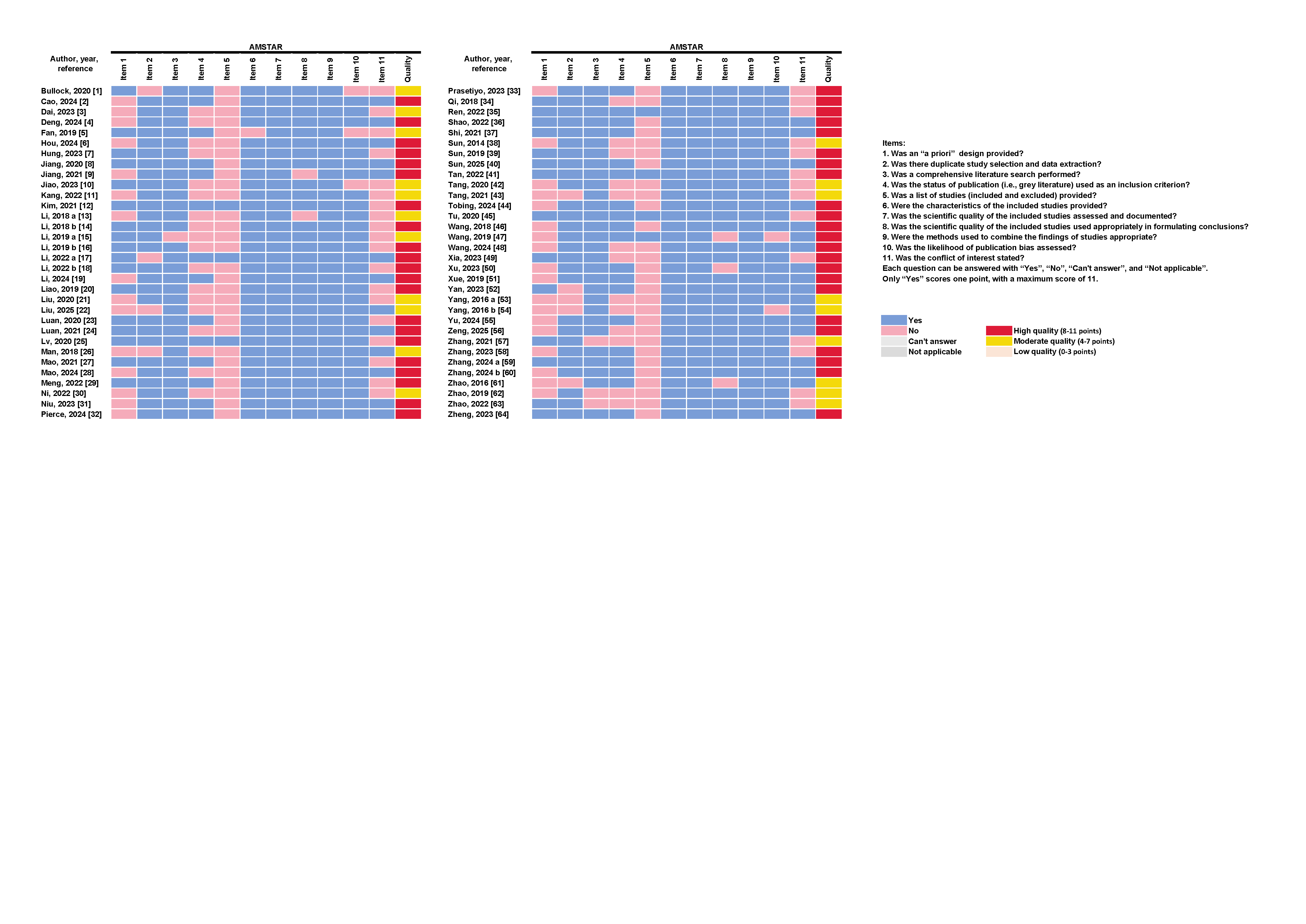
**Supplementary figure 1. Methodological quality assessment of the included articles with AMSTAR**

**References**

1. Bullock AF, Greenley SL, McKenzie GAG, Paton LW, Johnson MJ. Relationship between markers of malnutrition and clinical outcomes in older adults with cancer: systematic review, narrative synthesis and meta-analysis. *Eur J Clin Nutr* 2020; **74**(11): 1519-35.

2. Cao D, Dong Q. Predictive value of prognostic nutritional index for outcomes of cervical cancer: A systematic review and meta‑analysis. *Exp Ther Med* 2024; **28**(2): 316.

3. Dai M, Sun Q. Prognostic and clinicopathological significance of prognostic nutritional index (PNI) in patients with oral cancer: a meta-analysis. *Aging (Albany NY)* 2023; **15**(5): 1615-27.

4. Deng H, He Y, Huang G, Huang Y, Wu J, Qin X. Predictive value of prognostic nutritional index in patients undergoing gastrectomy for gastric cancer: A systematic review and meta-analysis. *Medicine (Baltimore)* 2024; **103**(41): e39917.

5. Fan X, Chen G, Li Y, et al. The Preoperative Prognostic Nutritional Index in Hepatocellular Carcinoma After Curative Hepatectomy: A Retrospective Cohort Study and Meta-Analysis. *Journal of investigative surgery : the official journal of the Academy of Surgical Research* 2019; **34**(8): 826-33.

6. Hou S, Song D, Hao R, Li L, Zhang Y, Zhu J. Prognostic relevance of prognostic nutritional indices in gastric or gastro-esophageal junction cancer patients receiving immune checkpoint inhibitors: a systematic review and meta-analysis. *Front Immunol* 2024; **15**: 1382417.

7. Hung KC, Sun CK, Chang YP, et al. Association of prognostic nutritional index with prognostic outcomes in patients with glioma: a meta-analysis and systematic review. *Front Oncol* 2023; **13**: 1188292.

8. Jiang AM, Zhao R, Liu N, et al. The prognostic value of pretreatment prognostic nutritional index in patients with small cell lung cancer and it's influencing factors: a meta-analysis of observational studies. *J Thorac Dis* 2020; **12**(10): 5718-28.

9. Jiang Y, Xu D, Song H, et al. Inflammation and nutrition-based biomarkers in the prognosis of oesophageal cancer: a systematic review and meta-analysis. *BMJ Open* 2021; **11**(9): e048324.

10. Jiao H, Wang L, Zhou X, Wu J, Li T. Prognostic Ability of Nutritional Indices for Outcomes of Bladder Cancer: A Systematic Review and Meta-Analysis. *Urol Int* 2023; **107**(9): 886-94.

11. Kang N, Gu H, Ni Y, Wei X, Zheng S. Prognostic and clinicopathological significance of the Prognostic Nutritional Index in patients with gastrointestinal stromal tumours undergoing surgery: a meta-analysis. *BMJ Open* 2022; **12**(12): e064577.

12. Kim SI, Kim SJ, Kim SJ, Cho DS. Prognostic nutritional index and prognosis in renal cell carcinoma: A systematic review and meta-analysis. *Urol Oncol* 2021; **39**(10): 623-30.

13. Li J, Xu R, Hu DM, Zhang Y, Gong TP, Wu XL. Prognostic Nutritional Index Predicts Outcomes of Patients after Gastrectomy for Cancer: A Systematic Review and Meta-Analysis of Nonrandomized Studies. *Nutr Cancer* 2018; **71**(4): 557-68.

14. Li D, Yuan X, Liu J, Li C, Li W. Prognostic value of prognostic nutritional index in lung cancer: a meta-analysis. *J Thorac Dis* 2018; **10**(9): 5298-307.

15. Li S, Tian G, Chen Z, Zhuang Y, Li G. Prognostic Role of the Prognostic Nutritional Index in Pancreatic Cancer: A Meta-analysis. *Nutr Cancer* 2019; **71**(2): 207-13.

16. Li P, Wang X, Lai Y, Zhou K, Tang Y, Che G. The prognostic value of pre-treatment prognostic nutritional index in esophageal squamous cell carcinoma: A meta-analysis. *Medicine (Baltimore)* 2019; **98**(22): e15280.

17. Li Z, Zhang D, Mo C, Zhu P, Fan X, Tang T. The prognostic significance of prognostic nutritional index in gastrointestinal stromal tumors: A systematic review and meta-analysis. *Medicine (Baltimore)* 2022; **101**(47): e32067.

18. Li P, Lai Y, Tian L, Zhou Q. The prognostic value of prognostic nutritional index in advanced cancer receiving PD-1/L1 inhibitors: A meta-analysis. *Cancer Med* 2022; **11**(16): 3048-56.

19. Li C, Yin Y, Yang Z, Zhang Q, Wang W, Liu J. Prognostic effect of the pretreatment prognostic nutritional index in cervical, ovarian, and endometrial cancer: a meta-analysis. *BMC Womens Health* 2024; **24**(1): 464.

20. Liao G, Zhao Z, Yang H, Chen M, Li X. Can Prognostic Nutritional Index be a Prediction Factor in Esophageal Cancer?: A Meta-Analysis. *Nutr Cancer* 2019; **72**(2): 187-93.

21. Liu M, Wang L. Prognostic significance of preoperative serum albumin, albumin-to-globulin ratio, and prognostic nutritional index for patients with glioma: A meta-analysis. *Medicine (Baltimore)* 2020; **99**(27): e20927.

22. Liu Y, Li K. Predictive role of the prognostic nutritional index for long-term prognosis among patients undergoing pancreatoduodenectomy: a meta-analysis. *BMC Surg* 2025; **25**(1): 51.

23. Luan C, Wang F, Wei N, Chen B. Prognostic nutritional index and the prognosis of diffuse large b-cell lymphoma: a meta-analysis. *Cancer Cell Int* 2020; **20**: 455.

24. Luan CW, Tsai YT, Yang HY, Chen KY, Chen PH, Chou HH. Pretreatment prognostic nutritional index as a prognostic marker in head and neck cancer: a systematic review and meta-analysis. *Sci Rep* 2021; **11**(1): 17117.

25. Lv X, Zhang Z, Yuan W. Pretreatment Prognostic Nutritional Index (PNI) as a Prognostic Factor in Patients with Biliary Tract Cancer: A Meta-Analysis. *Nutr Cancer* 2020; **73**(10): 1872-81.

26. Man Z, Pang Q, Zhou L, et al. Prognostic significance of preoperative prognostic nutritional index in hepatocellular carcinoma: a meta-analysis. *HPB (Oxford)* 2018; **20**(10): 888-95.

27. Mao C, Xu W, Ma W, Wang C, Guo Z, Yan J. Prognostic Value of Pretreatment Prognostic Nutritional Index in Patients With Renal Cell Carcinoma: A Meta-Analysis. *Front Oncol* 2021; **11**: 719941.

28. Mao S, Zhang Z, Li Y. Prognostic and clinicopathological role of prognostic nutritional index (PNI) in endometrial cancer: A meta-analysis. *Heliyon* 2024; **10**(15): e35211.

29. Meng C, Gan L, Li K, et al. Prognostic nutritional index before surgical treatment may serve as a prognostic biomarker for patients with upper tract urothelial carcinoma: A systematic review and meta-analysis. *Front Nutr* 2022; **9**: 972034.

30. Ni L, Huang J, Ding J, et al. Prognostic Nutritional Index Predicts Response and Prognosis in Cancer Patients Treated With Immune Checkpoint Inhibitors: A Systematic Review and Meta-Analysis. *Front Nutr* 2022; **9**: 823087.

31. Niu Z, Yan B. Prognostic and clinicopathological effect of the prognostic nutritional index (PNI) in patients with cervical cancer: a meta-analysis. *Ann Med* 2023; **55**(2): 2288705.

32. Pollock M, Fernandes RM, Pieper D, et al. Preferred Reporting Items for Overviews of Reviews (PRIOR): a protocol for development of a reporting guideline for overviews of reviews of healthcare interventions. *Syst Rev* 2019; **8**(1): 335.

33. Prasetiyo PD, Baskoro BA, Hariyanto TI. The role of nutrition-based index in predicting survival of breast cancer patients: A systematic review and meta-analysis. *Heliyon* 2023; **10**(1): e23541.

34. Qi F, Zhou X, Wang Y, et al. Pre-treatment prognostic nutritional index may serve as a potential biomarker in urinary cancers: a systematic review and meta-analysis. *Cancer Cell Int* 2018; **18**: 207.

35. Ren W, Wang H, Xiang T, Liu G. Prognostic Role of Preoperative Onodera's Prognostic Nutritional Index (OPNI) in Gastrointestinal Stromal Tumors: a Systematic Review and Meta-analysis. *J Gastrointest Cancer* 2022; **54**(3): 731-8.

36. Shao Y, Cao W, Gao X, Tang M, Zhu D, Liu W. Pretreatment "prognostic nutritional index" as an indicator of outcome in lung cancer patients receiving ICI-based treatment: Systematic review and meta-analysis. *Medicine (Baltimore)* 2022; **101**(43): e31113.

37. Shi Y, Zhang Y, Niu Y, Chen Y, Kou C. Prognostic role of the prognostic nutritional index (PNI) in patients with head and neck neoplasms undergoing radiotherapy: A meta-analysis. *PLoS One* 2021; **16**(9): e0257425.

38. Sun K, Chen S, Xu J, Li G, He Y. The prognostic significance of the prognostic nutritional index in cancer: a systematic review and meta-analysis. *J Cancer Res Clin Oncol* 2014; **140**(9): 1537-49.

39. Sun G, Li Y, Peng Y, et al. Impact of the preoperative prognostic nutritional index on postoperative and survival outcomes in colorectal cancer patients who underwent primary tumor resection: a systematic review and meta-analysis. *Int J Colorectal Dis* 2019; **34**(4): 681-9.

40. Sun J, Li Z, Zhu X. Prognostic role of prognostic nutritional index in patients with bladder cancer: a systematic review and meta-analysis. *Front Oncol* 2025; **14**: 1486389.

41. Tan X, Chen H. The Prognostic Value of Prognostic Nutritional Index in Patients with Ovarian Cancer: A Systematic Review and Meta-Analysis. *Nutr Cancer* 2022; **75**(1): 73-81.

42. Tang M, Jia Z, Zhang J. The prognostic role of prognostic nutritional index in nasopharyngeal carcinoma: A systematic review and meta-analysis. *Int J Clin Oncol* 2020; **26**(1): 66-77.

43. Tang Y, Liang J, Liu Z, et al. Clinical significance of prognostic nutritional index in renal cell carcinomas. *Medicine (Baltimore)* 2021; **100**(10): e25127.

44. Tobing E, Tansol C, Tania C, Sihombing AT. Prognostic Nutritional Index (PNI) as Independent Predictor of Poor Survival in Prostate Cancer: A Systematic Review and Meta-Analysis. *Clin Genitourin Cancer* 2024; **22**(5): 102142.

45. Tu X, Ren J, Zhao Y. Prognostic value of prognostic nutritional index in nasopharyngeal carcinoma: A meta-analysis containing 4511 patients. *Oral Oncol* 2020; **110**: 104991.

46. Wang Z, Wang Y, Zhang X, Zhang T. Pretreatment prognostic nutritional index as a prognostic factor in lung cancer: Review and meta-analysis. *Clin Chim Acta* 2018; **486**: 303-10.

47. Wang X, Wang Y. The prognostic nutritional index is prognostic factor of gynecological cancer: A systematic review and meta-analysis. *Int J Surg* 2019; **67**: 79-86.

48. Wang L, Long X, Zhu Y, Luo A, Yang M. Association of prognostic nutritional index with long-term survival in lung cancer receiving immune checkpoint inhibitors: A meta-analysis. *Medicine (Baltimore)* 2024; **103**(52): e41087.

49. Xia H, Zhang W, Zheng Q, et al. Predictive value of the prognostic nutritional index in advanced non-small cell lung cancer patients treated with immune checkpoint inhibitors: A systematic review and meta-analysis. *Heliyon* 2023; **9**(8): e17400.

50. Xu XT, Qian Y, Tian MX, et al. Predictive Impact of Prognostic Nutritional Index in Patients with Cancer Treated with Immune Checkpoint Inhibitors: A Systematic Review and Meta-Analysis. *Nutr Cancer* 2023; **75**(6): 1413-26.

51. Xue W, Xu X, Tan Y, et al. Evaluating and validating the predictive ability of preoperative systemic inflammatory/immune cells in gastric cancer following R0 resection. *Oncol Lett* 2019; **18**(5): 5205-14.

52. Yan X, Wang J, Mao J, et al. Identification of prognostic nutritional index as a reliable prognostic indicator for advanced lung cancer patients receiving immune checkpoint inhibitors. *Front Nutr* 2023; **10**: 1213255.

53. Yang Y, Gao P, Song Y, et al. The prognostic nutritional index is a predictive indicator of prognosis and postoperative complications in gastric cancer: A meta-analysis. *Eur J Surg Oncol* 2016; **42**(8): 1176-82.

54. Yang Y, Gao P, Chen X, et al. Prognostic significance of preoperative prognostic nutritional index in colorectal cancer: results from a retrospective cohort study and a meta-analysis. *Oncotarget* 2016; **7**(36): 58543-52.

55. Yu M, Li X, Chen M, et al. Prognostic potential of nutritional risk screening and assessment tools in predicting survival of patients with pancreatic neoplasms: a systematic review. *Nutr J* 2024; **23**(1): 17.

56. Zeng D, Wen NY, Wang YQ, Cheng NS, Li B. Prognostic roles nutritional index in patients with resectable and advanced biliary tract cancers. *World J Gastroenterol* 2025; **31**(6): 97697.

57. Zhang Q, Bao J, Zhu ZY, Jin MX. Prognostic nutritional index as a prognostic factor in lung cancer patients receiving chemotherapy: a systematic review and meta-analysis. *Eur Rev Med Pharmacol Sci* 2021; **25**(18): 5636-52.

58. Zhang L, Ma W, Qiu Z, et al. Prognostic nutritional index as a prognostic biomarker for gastrointestinal cancer patients treated with immune checkpoint inhibitors. *Front Immunol* 2023; **14**: 1219929.

59. Zhang H, Li D, Li J. Prognostic significance of preoperative prognostic nutritional index in hepatocellular carcinoma after curative hepatectomy: a meta-analysis and systemic review. *Front Nutr* 2024; **11**: 1433528.

60. Zhang L, Wang F, Wan C, Tang J, Qin J. Prognostic Nutritional Index and the Survival of Patients with Endometrial cancer: A Meta-analysis. *Reproductive sciences (Thousand Oaks, Calif)* 2024; **31**(12): 3779-94.

61. Zhao Y, Xu P, Kang H, et al. Prognostic nutritional index as a prognostic biomarker for survival in digestive system carcinomas. *Oncotarget* 2016; **7**(52): 86573-83.

62. Zhao J, Chen C, Cheng J, et al. Low pretreatment prognostic nutritional index predicts poor survival of urologic cancer: an observational meta-analysis. *International Journal of Clinical and Experimental Medicine* 2019; **12**(5): 4540-9.

63. Zhao P, Wu Z, Wang Z, Wu C, Huang X, Tian B. Prognostic role of the prognostic nutritional index in patients with pancreatic cancer who underwent curative resection without preoperative neoadjuvant treatment: A systematic review and meta-analysis. *Frontiers in surgery* 2022; **9**: 992641.

64. Zheng Y, Wang K, Ou Y, et al. Prognostic value of a baseline prognostic nutritional index for patients with prostate cancer: a systematic review and meta-analysis. *Prostate Cancer Prostatic Dis* 2023; **27**(4): 604-13.

65. Feng YW, Wang HY, Lin Q. Can the preoperative prognostic nutritional index be used as a postoperative predictor of gastric or gastroesophageal junction adenocarcinoma? *World J Gastrointest Oncol* 2024; **16**(7): 2877-80.

66. Fiflis S, Christodoulidis G, Papakonstantinou M, et al. Prognostic nutritional index in predicting survival of patients with gastric or gastroesophageal junction adenocarcinoma: A systematic review. *World J Gastrointest Oncol* 2024; **16**(2): 514-26.

67. Grimes N, Tyson M, Hannan C, Mulholland C. A Systematic Review of the Prognostic Role of Hematologic Scoring Systems in Patients With Renal Cell Carcinoma Undergoing Nephrectomy With Curative Intent. *Clin Genitourin Cancer* 2016; **14**(4): 271-6.

68. Laszkiewicz J, Krajewski W, Sojka A, et al. Blood-, Tissue- and Urine-Based Prognostic Biomarkers of Upper Tract Urothelial Carcinoma. *Diagnostics (Basel)* 2024; **14**(17).

69. Li CJ, Lee PC, Huang KW, et al. Postoperative prognostic nutrition index predicts survival in patients with small bowel adenocarcinoma after surgical resection. *Journal of the Chinese Medical Association : JCMA* 2024; **87**(9): 819-27.

70. Morelli I, Greto D, Visani L, et al. Integrating nutritional status and hematological biomarkers for enhanced prognosis prediction in glioma patients: A systematic review. *Clin Nutr ESPEN* 2025; **66**: 269-80.

71. Teja M, Garrido MI, Ocanto A, Counago F. Prognostic impact of inflammatory and nutritional biomarkers in pancreatic cancer. *World J Clin Oncol* 2025; **16**(1): 101191.

72. Yan L, Nakamura T, Casadei-Gardini A, Bruixola G, Huang YL, Hu ZD. Long-term and short-term prognostic value of the prognostic nutritional index in cancer: a narrative review. *Ann Transl Med* 2021; **9**(21): 1630.
